# Genetic and functional data identifying *Cd101* as a type 1 diabetes (T1D) susceptibility gene in nonobese diabetic (NOD) mice

**DOI:** 10.1371/journal.pgen.1008178

**Published:** 2019-06-14

**Authors:** Jochen Mattner, Javid P. Mohammed, Michael E. Fusakio, Claudia Giessler, Carl-Philipp Hackstein, Robert Opoka, Marius Wrage, Regina Schey, Jan Clark, Heather I. Fraser, Daniel B. Rainbow, Linda S. Wicker

**Affiliations:** 1 Mikrobiologisches Institut—Klinische Mikrobiologie, Immunologie und Hygiene, Universitätsklinikum Erlangen and Friedrich-Alexander Universität Erlangen-Nürnberg, Erlangen, Germany; 2 Division of Immunobiology, Cincinnati Children's Hospital, Cincinnati, OH, United States of America; 3 JDRF/Wellcome Trust Diabetes and Inflammation Laboratory, Wellcome Trust/MRC Building, Cambridge Institute for Medical Research, NIHR Cambridge Biomedical Research Centre, University of Cambridge, Cambridge, United Kingdom; 4 JDRF/Wellcome Trust Diabetes and Inflammation Laboratory, Wellcome Trust Centre for Human Genetics, Nuffield Department of Medicine, National Institute for Health Research (NIHR) Oxford Biomedical Research Centre, University of Oxford, Oxford, United Kingdom; The Jackson Laboratory, UNITED STATES

## Abstract

Type 1 diabetes (T1D) is a chronic multi-factorial disorder characterized by the immune-mediated destruction of insulin-producing pancreatic beta cells. Variations at a large number of genes influence susceptibility to spontaneous autoimmune T1D in non-obese diabetic (NOD) mice, one of the most frequently studied animal models for human disease. The genetic analysis of these mice allowed the identification of many insulin-dependent diabetes (*Idd*) loci and candidate genes, one of them being *Cd101*. CD101 is a heavily glycosylated transmembrane molecule which exhibits negative-costimulatory functions and promotes regulatory T (Treg) function. It is abundantly expressed on subsets of lymphoid and myeloid cells, particularly within the gastrointestinal tract. We have recently reported that the genotype-dependent expression of CD101 correlates with a decreased susceptibility to T1D in NOD.B6 *Idd10* congenic mice compared to parental NOD controls. Here we show that the knockout of CD101 within the introgressed B6-derived *Idd10* region increased T1D frequency to that of the NOD strain. This loss of protection from T1D was paralleled by decreased Gr1-expressing myeloid cells and FoxP3^+^ Tregs and an enhanced accumulation of CD4-positive over CD8-positive T lymphocytes in pancreatic tissues. As compared to CD101^+/+^ NOD.B6 *Idd10* donors, adoptive T cell transfers from CD101^−/−^ NOD.B6 *Idd10* mice increased T1D frequency in lymphopenic NOD *scid* and NOD.B6 *Idd10 scid* recipients. Increased T1D frequency correlated with a more rapid expansion of the transferred CD101^−/−^ T cells and a lower proportion of recipient Gr1-expressing myeloid cells in the pancreatic lymph nodes. Fewer of the Gr1^+^ cells in the recipients receiving CD101^−/−^ T cells expressed CD101 and the cells had lower levels of IL-10 and TGF-β mRNA. Thus, our results connect the *Cd101* haplotype-dependent protection from T1D to an anti-diabetogenic function of CD101-expressing Tregs and Gr1-positive myeloid cells and confirm the identity of *Cd101* as *Idd10*.

## Author summary

The complex interplay of environmental factors and genetic traits determines the susceptibility of an individual to autoimmune disease such as type 1 diabetes (T1D). Despite T1D being one of the most common and most studied polygenic autoimmune disorders, the mechanisms underlying the immune-mediated destruction of the insulin-producing pancreatic beta cells are still largely unknown. Genetic association studies identified many DNA sequence variants that confer risk to or protect from autoimmune disease. In this regard, we have identified a single gene, *Cd101*, as a T1D susceptibility locus. In accordance with our previous studies in which we reported an association of allelic *Cd101* variants on T1D prevalence, we observed here that deletion of *Cd101* perpetuated the expansion of pathogenic, pancreas-infiltrating immune cells and subsequently enhanced T1D incidence. The mechanisms by which *Cd101* variants interfere with autoimmune responses will allow us to understand the regulation of molecules in autoimmunity in general as diabetes susceptibility loci have been associated with other autoimmune diseases. Consequently, our work will help to identify therapeutic approaches that can be used to guide the development of effective therapies for T1D, but also allows the identification of common targets in autoimmune disease for clinical intervention in the future.

## Introduction

Type I diabetes (T1D) is a complex autoimmune disease driven by multiple genetic traits and facilitated by various immune cells infiltrating the pancreatic islets. In rodent models such as the frequently studied non-obese diabetic (NOD) mouse, myeloid cells including macrophages, dendritic cells (DCs) and neutrophils are the first cells to accumulate in the pancreas [1–4] and contribute to the initiation and perpetuation of the T cell-driven pancreatic islet destruction [5]. Myeloid-derived suppressor cells (MDSCs) and regulatory T cells (Tregs), in contrast, suppress diabetogenic immune cells and hinder the development of T1D [6, 7]. While the transcription factor FoxP3 identifies Tregs, the delineation of MDSCs from other myeloid cell subsets is challenging and requires a scrutinized investigation.

Tregs are pivotal for the maintenance of immune homeostasis. Their ability to suppress other immune cells maintains tolerance to self-antigens and prevents autoimmune disease. The depletion of Tregs promotes the development of T1D while their transfer or therapeutic enhancement exhibits protective effects [8–10]. Compared to other reference strains, some studies reported primary deficits in Treg numbers of NOD mice [8, 11–13], whereas others did not [14–19]. Although Treg frequencies in T1D patients appear normal in most studies, defects in the phenotype and the suppressive capacity of Tregs have been reported [20–23]. In mice, induced NOD-derived Tregs are also less effective in standard *in vitro* suppression assays and reveal subtle defects in the expression of distinct genes [18], although the relevance of these genes on Treg function and on the induction of T1D in NOD mice remains to be determined. An age-related decline in Treg function of NOD mice over time is also pivotal for T1D development in NOD mice [14–16].

Various myeloid cell populations and myeloid cell responses are frequently altered and impeded in NOD mice [24]. Thus, the development of precursors to dendritic cells (DCs) and macrophages, for example, is hampered in NOD mice [25–27] as well as the maturation of myeloid DCs [28]. In addition, the recruitment of neutrophils to sites of infection is severely impaired [24]. The molecular signals underlying these phenotypes, however, have rarely been identified.

CD101 is a negative costimulatory molecule expressed on subsets of myeloid and lymphoid cells [29–33]. Upon engagement of CD101 by agonistic antibodies myeloid cells are induced to be immunosuppressive *in vitro* [34]. *In vitro*, CD101^+^ Tregs are more suppressive than their CD101^−^ counterparts [30]. *In vivo*, CD101 perpetuates the suppressive function of Tregs and reduces the development of T1D and chronic colitis [30, 31, 35]. Furthermore, CD101^+^ myeloid cells release more IL-10 than CD101^−^ myeloid cells [35]. In addition, reduced CD101 expression is observed in T1D and Inflammatory Bowel Disease patients [35, 36]. Rare polymorphisms in the *Cd101* gene have been suggested to underlie the reduced CD101 expression in some T1D patients [36].

We have identified *Cd101* as a T1D candidate gene within the *Idd10* region using multiple *Idd10* congenic strains [37]. Susceptibility to T1D was correlated with genotype-dependent CD101 expression on multiple cell subsets, including Foxp3^+^ Tregs, CD11c^+^ dendritic cells, and Gr1^+^ myeloid cells [31]. To evaluate the impact of CD101 on T1D, we introgressed the *Idd10* and *Idd10/Idd18* regions from a B6 CD101 KO strain onto the NOD background and observed that T1D protection mediated by the B6-derived *Idd10* and *Idd10/Idd18* regions was lost in CD101^−/−^ NOD.B6 *Idd10* and CD101^−/−^ NOD.B6 *Idd10/Idd18* mice. The loss of CD101 expression reduced the frequency of Tregs and transformed anti-inflammatory Gr1-expressing myeloid cells into an inflammatory, disease-promoting subset. Thus, our data further confirm the identity of *Cd101* as *Idd10* and provide cellular mechanisms by which the molecule mediates its protection from T1D.

## Results

### Expansion of CD101-expressing regulatory T cells in the pancreatic lymph nodes of NOD mice carrying the B6 *Cd101* allele

**Fig 1:**
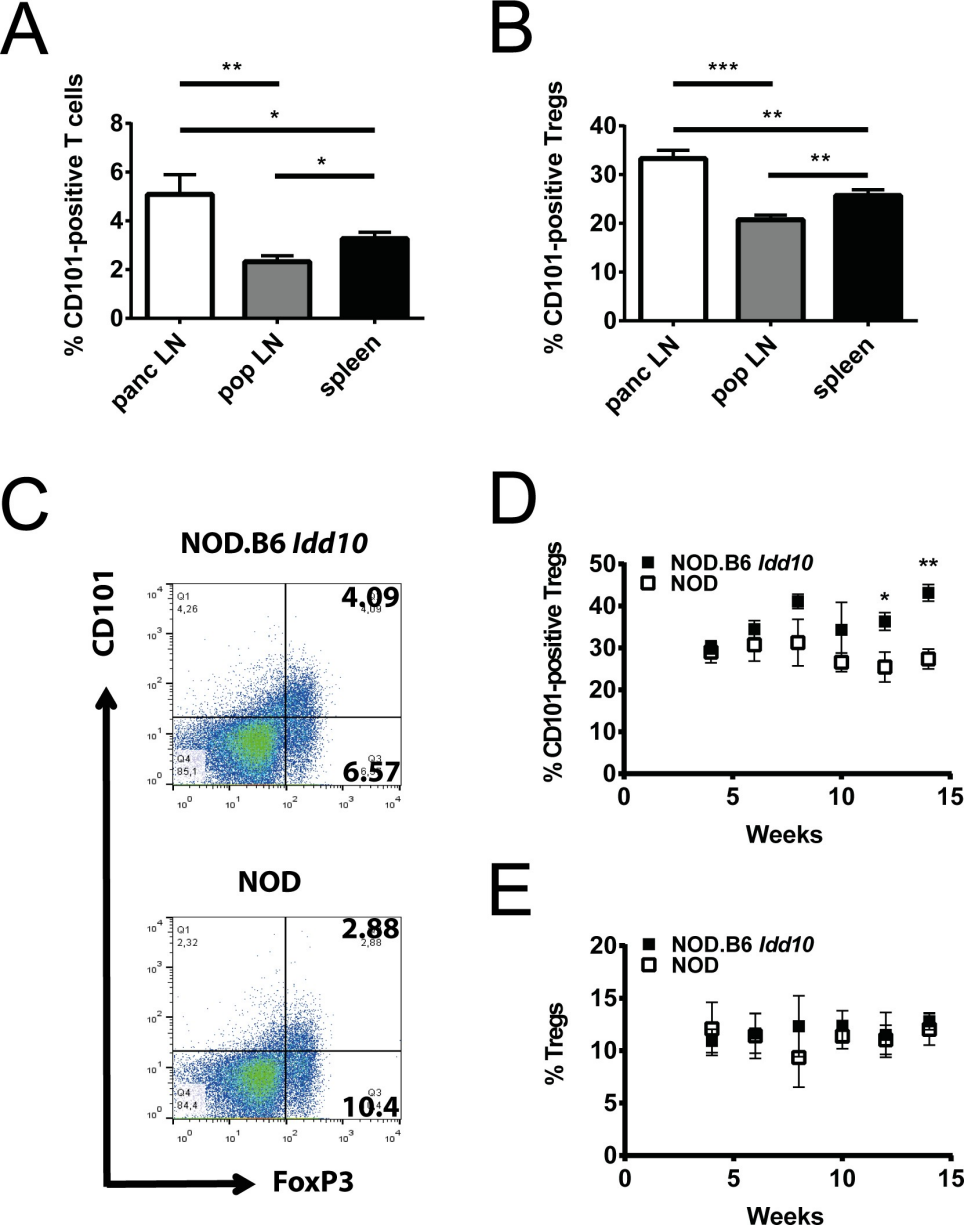
CD101-positive T cells accumulate in the pancreatic lymph nodes of NOD.B6 *Idd10* mice. The expression of CD101 on TCRβ-positive T cells (A) and FoxP3-positive Tregs (B-E) was assessed by flow cytometry in the spleens (A, B), pancreatic (panc) (A-E) and popliteal (pop) (A, B) lymph nodes. The compiled data of five individual 4-8-week-old NOD.B6 *Idd10* mice (A, B) are displayed. Representative FACS plots are shown in S2 Fig. The expression of FoxP3 and CD101 was assessed on T lymphocytes expressing the β-chain of the TCR and CD4 (C-E). Representative FACS plots for NOD.B6 *Idd10* (upper panel) and NOD (lower panel) mice are displayed (C). The percentage of CD101-expressing Tregs (D) as well as the percentage of FoxP3-positive Tregs (E) was compiled of 4 (week 4, 10, 12 and 14 of age) or 5 (week 6 and 8) individual mice per time point. Comparisons between groups were performed using the Mann–Whitney nonparametric test (*, p<0.05; **, p<0.01; ***, p<0.001). Error bars indicate the SD of the mean.

Auto-reactive T cells are initially primed in the pancreatic lymph nodes of NOD mice beginning at the age of three weeks [5] and attracted by an infiltration of innate immune cells into the pancreatic islets one to three weeks later [38]. As we had observed that CD101 expression was genotype-dependent in bone marrow and spleen in NOD *Idd10* congenic strains [31], we assessed the influence of *Cd101* gene variation on the distribution of immune cells in peripheral lymph nodes as compared to spleen. In both lymph nodes and spleen CD101 expression was observed on a portion of T cells and myeloid cells (Fig 1A, S1A and S1B Fig). T cells constituted the majority of CD101-expressing cells in peripheral lymph nodes but not in the spleen (S1C Fig). A small portion of the CD101-expressing cells were neither T cells nor CD11b^+^ (S1C Fig). We observed an increased proportion of CD101-positive T cells and of CD101-positive Tregs which represent the largest CD101-positive subset within the T lymphocyte population [29–33] in the pancreatic lymph nodes of 4-8-week-old NOD and NOD.B6 *Idd10* mice compared to other peripheral lymph nodes (Fig 1A and 1B; S2 Fig, S3 Fig). The percentages of CD101-positive T cells between NOD and NOD.B6 *Idd10* mice were comparable (S3B Fig) while the mean fluorescence intensity for CD101 was often enhanced on T cells from NOD.B6 *Idd10* mice (S3C Fig), a phenotype observed previously in splenic T cells [31]. CD101-expressing Tregs in pancreatic, but not popliteal, lymph nodes of NOD.B6 *Idd10* mice were more frequent at the two later time points assessed compared to NOD mice (Fig 1C and 1D; S4A Fig), while Treg frequencies themselves in both organs remained comparable (Fig 1C and 1E; S4B Fig). The proportion of other CD101-expressing myeloid and lymphoid subsets was also similar between NOD and NOD.B6 *Idd10* mice (S5 Fig). Thus, the introgression of the B6 *Idd10* region not only promotes the expansion of Gr1-positive myeloid cells in the bone marrow [31] but also favors at later time-points an accumulation of CD101-expressing Tregs in the pancreatic lymph nodes.

### Generation of CD101^−/−^ NOD.B6 *Idd10* mice and CD101^−/−^ NOD.B6 *Idd10/18* mice

**Fig 2:**
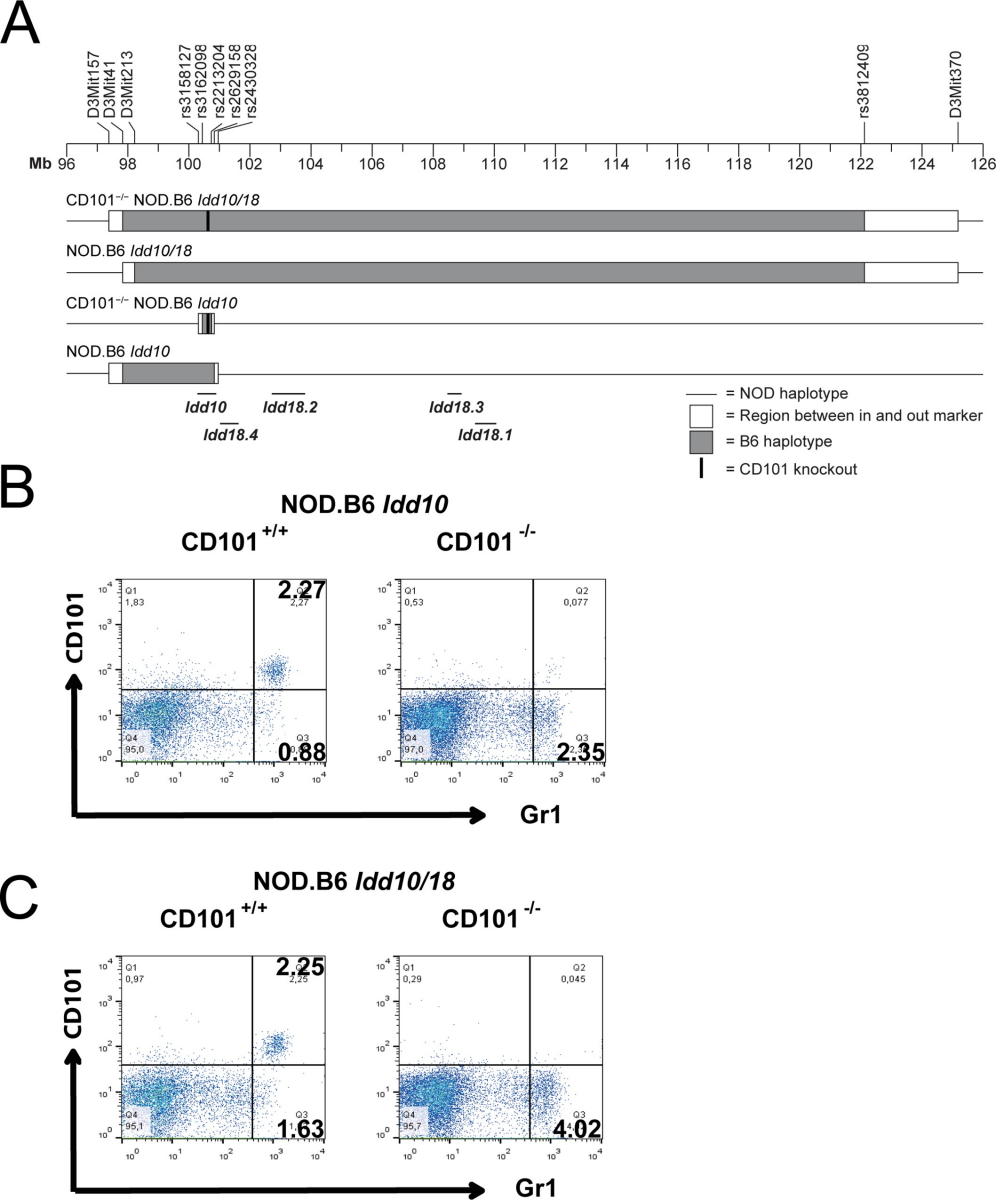
Deletion of *Cd101* exon 1 leads to a loss of protein expression. Representation of the *Idd10* and *Idd18* regions and the NOD congenic strains used in this study (A). Lack of CD101 expression in CD101^−/−^ NOD.B6 *Idd10* (B) and CD101^−/−^ NOD.B6 *Idd10/18* mice (C). CD11b-positive cells in the spleens of CD101^−/−^ NOD.B6 *Idd10* (B) and CD101^−/−^ NOD.B6 *Idd10/18* mice (C) as well as CD101-expressing control littermates were evaluated for their CD101-expression by flow cytometry. Representative FACS dot plots are displayed.

Based on sequence comparisons of four *Idd10* regions tested for T1D susceptibility and the observation that susceptibility to T1D correlated with genotype-dependent CD101 expression on multiple immune cell subsets, *Cd101* is the prioritized gene candidate for the *Idd10* region [31]. We therefore reasoned that if *Cd101* is *Idd10*, elimination of CD101 protein expression should alter T1D susceptibility, a finding that would further strengthen our hypothesis that allelic variation in the structure or expression of CD101 influences T1D frequency in the context of the NOD background. Following deletion of a portion of the B6 *Cd101* gene required for protein expression [31] the T1D-protective *Idd10* and *Idd10/18* regions carrying the *Cd101* modification were introgressed onto the NOD background by genotype-selected backcrossing to generate congenic strains (Fig 2A). We wanted to examine the effect of eliminating CD101 expression both in the context of the B6-derived protective *Idd10* region, but also in the context of the more complex *Idd10/18* region where multiple *Idd* subregions have been defined [39, 40] and are depicted on Fig 2A. Therefore, as backcrossing of the B6 CD101^−/−^ strain to the NOD strain occurred, we screened progeny for recombination events that most closely resembled those defining the boundaries of the regions in previous studies in order to later characterize the strains versus the congenic regions with intact B6 *Cd101* alleles. As was observed on the B6 background [31], the engineered deletion within the *Cd101* gene led to a lack of CD101 protein expression on all myeloid and T cells in CD101^−/−^ NOD.B6 *Idd10* (Fig 2B; S2 Fig) and CD101^−/−^ NOD.B6 *Idd10/18* mice (Fig 2C).

### The genetic deletion of CD101 within the *Idd10* region abolishes the protection from T1D in NOD.B6 *Idd10* mice

**Fig 3:**
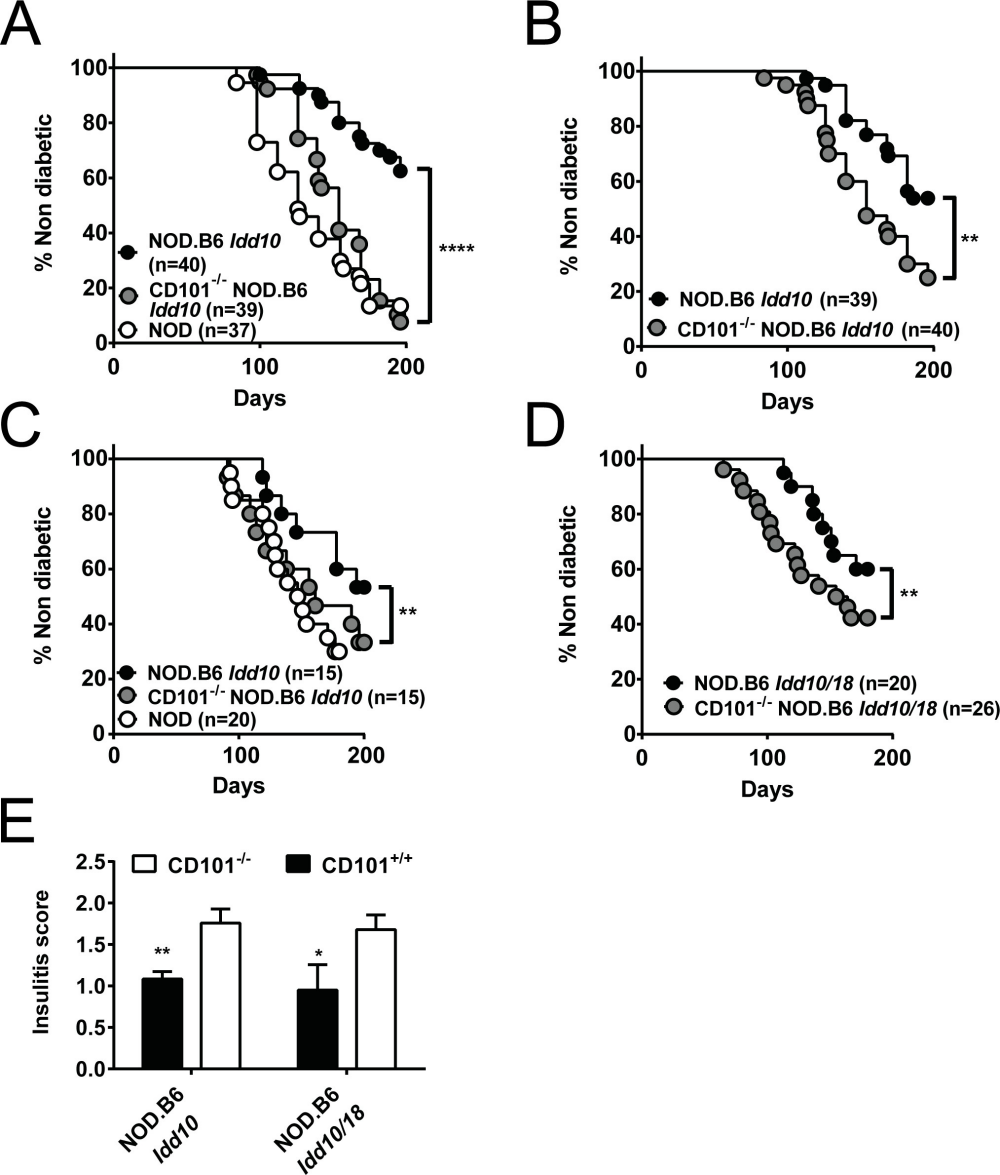
CD101-expression protects NOD.B6 *Idd10* and NOD.B6 *Idd10/18* mice from T1D. The frequency of T1D was assessed by the analysis of urinary glucose concentration (A-D) and histopathologic lesions (E) in the indicated number of females in parental NOD mice, CD101^+/+^ NOD.B6 *Idd10* mice and CD101^−/−^ NOD.B6 *Idd10* mice (A-C) as well as in CD101^+/+^ NOD.B6 *Idd10/18* mice and CD101^−/−^ NOD.B6 *Idd10/18* mice (D). Mice were derived from homozygous breeders except in the case of mice shown in (B) where progeny of heterozygous breeders were monitored for T1D. T1D incidence was assessed at Taconic (A+B, performed contemporaneously so the NOD control group shown in A is also the NOD control group for the groups of mice shown in B) and at CCHMC (C+D, performed contemporaneously so the NOD control group shown in C is also the NOD control group for the groups of mice shown in D). Histopathologic analysis of pancreata was performed in nondiabetic 8–10 week-old CD101^−/−^ NOD.B6 *Idd10* (n = 5), CD101^+/+^ NOD.B6 *Idd10* (n = 6), CD101^−/−^ NOD.B6 *Idd10/Idd18* (n = 5) and CD101^+/+^ NOD.B6 *Idd10/Idd18* (n = 4) female mice (E). For T1D frequency comparisons Kaplan–Meier survival curves were plotted for each mouse strain, and statistical significance was determined by log rank test (*, p<0.05; **, p<0.01; ****, p<0.0001). For insulitis comparisons a one-tailed t-text was used (*, p<0.05; **, p<0.01). Error bars indicate the SD of the mean.

We have recently reported a correlation of CD101 expression on immune cells from four independent *Idd10* haplotypes with the development of T1D [31]. Thus, to further establish the causative role of *Cd101* in the pathogenesis of T1D, we evaluated T1D frequencies in our newly generated CD101^−/−^ NOD.B6 *Idd10* and CD101^−/−^ NOD.B6 *Idd10/18* mice compared to parental CD101-expressing NOD.B6 *Idd10* and NOD.B6 *Idd10/18* controls. We observed that CD101-expressing NOD.B6 *Idd10* and NOD.B6 *Idd10/18* mice developed T1D substantially slower and with a reduced incidence than their CD101-deficient counterparts in two different animal facilities (Fig 3). Indeed, CD101^−/−^ NOD.B6 *Idd10* mice and CD101^−/−^ NOD.B6 *Idd10/18* mice had frequencies of diabetes equivalent to that of NOD mice housed in the same colony (Fig 3A, 3C and 3D). Contemporaneously, cohorts of CD101^+/+^ and CD101^−/−^ NOD.B6 *Idd10* mice as well as CD101^+/+^ and CD101^−/−^ NOD.B6 *Idd10* progeny from heterozygous CD101^+/−^ NOD.B6 *Idd10* intercross breeders were monitored for diabetes (Fig 3A and 3B). In the latter comparison CD101-replete and CD101 KO *Idd10* homozygous progeny are part of the same litters and therefore exposed to the same micro-environment. Once again, mice homozygous for the CD101 KO *Idd10* region had a higher frequency of diabetes than those having two doses of the intact B6-derived *Idd10* region (Fig 3B); however, the difference in diabetes occurrence was less significant between the homozygous genotypes derived from heterozygous breeders than when the mice had been bred from homozygous parents (Fig 3A). CD101^+/−^ NOD.B6 *Idd10* heterozygous progeny had a diabetes frequency intermediate between those of CD101^+/+^ and CD101^−/−^ NOD.B6 *Idd10* progeny (S6 Fig). Protection from diabetes was associated with a significantly reduced infiltration of pancreatic islets by immune cells in CD101-expressing congenic mice at 8–10 weeks of age (Fig 3E, S7 Fig). Thus, these data confirm the protective role of the B6 *Cd101* allele within the *Idd10* region and strongly suggest that the gene encoding CD101 is *Idd10*.

### Loss of protection from T1D in CD101^−/−^ NOD.B6 *Idd10* mice is associated with a decrease of Tregs and an overrepresentation of CD4- over CD8-positive T cells

**Fig 4:**
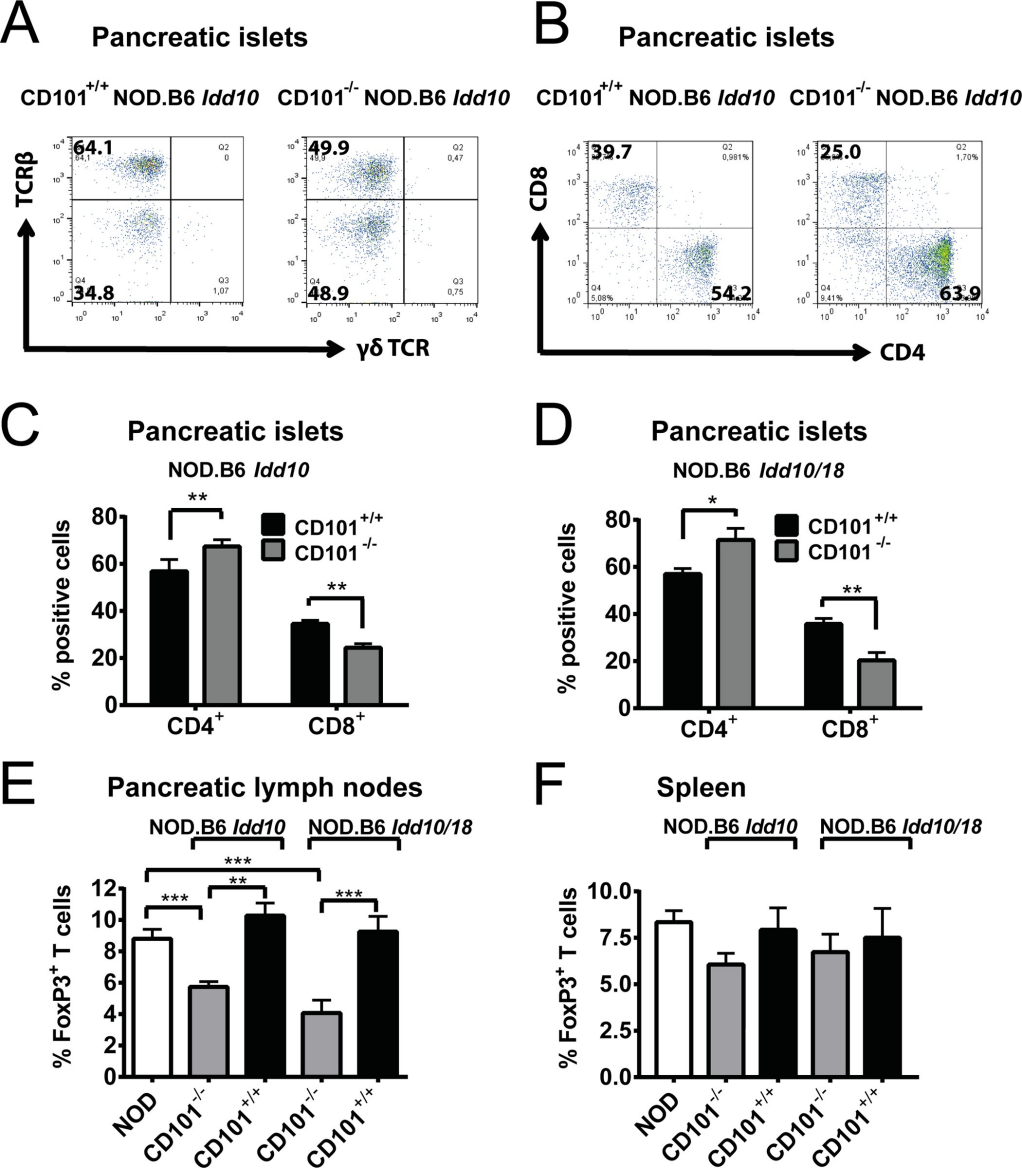
CD101-expression sustains CD8-positive T lymphocytes and Tregs. Representative profiles of inflammatory infiltrates in pancreatic islets of CD101^+/+^ and CD101^−/−^ NOD.B6 *Idd10* mice show that they consist mainly of TCRβ^+^ T lymphocytes (A). The accumulation of CD8- over CD4-positive T lymphocytes is enhanced in CD101-expressing NOD.B6 *Idd10* congenic mice (B). Representative FACS dot plots of T cells from pancreatic islets which were pre-gated on TCRβ and the summary of CD4- and CD8-positive T lymphocytes from five individual, 14 week-old female mice per strain are displayed (C+D). The expression of FoxP3 was assessed on lymphocytes expressing the β-chain of the TCR and CD4 in the pancreatic lymph nodes (E) and spleens (F) of the indicated mouse strains. The results of 13 individual, 10–13 week-old female mice were summarized from three independent experiments. Statistical significant differences were determined using Mann-Whitney tests (*, p<0.05; **, p<0.01; ***, p<0.001). Error bars indicate the SD of the mean.

**Fig 5:**
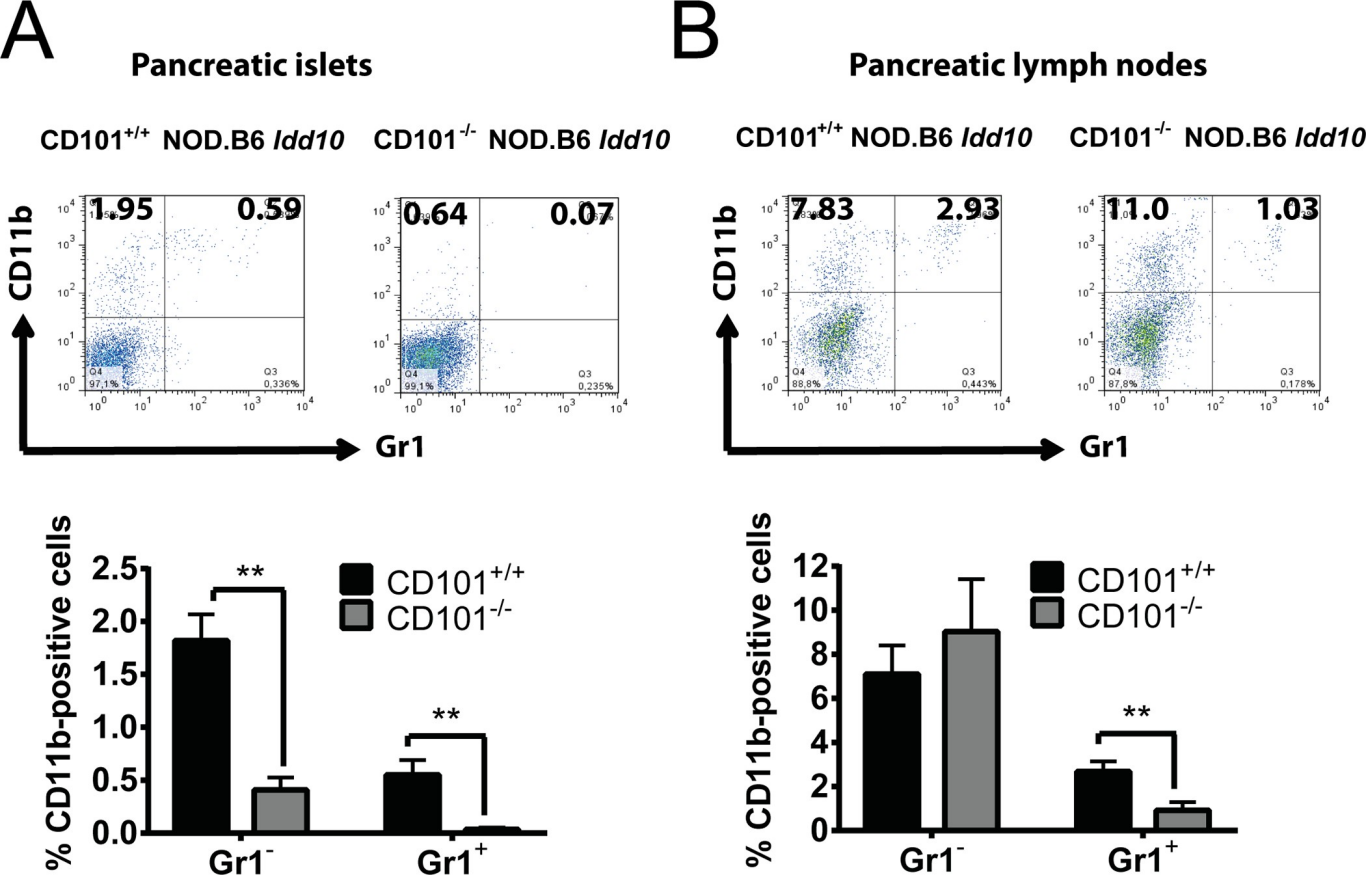
Gr1-expressing myeloid cells accumulate in the pancreatic lesions of CD101^+/+^ NOD.B6 *Idd10* mice. Representative FACS dot plots of Gr1- and/or CD11b-expressing myeloid cells from the pancreas (A) and the pancreatic lymph nodes (B) of 14-week-old CD101^+/+^ and CD101^−/−^ NOD.B6 *Idd10* mice are displayed (upper panels). The percentages of Gr1-positive and Gr1-negative cells among CD11b-expressing myeloid cells were compiled from 8 individual CD101^−/−^ NOD.B6 *Idd10* and CD101^+/+^ NOD.B6 *Idd10* mice (A+B, lower panels). Statistical significance was determined using Mann-Whitney tests (*, p<0.05; **, p<0.01; ***, p<0.001). Error bars indicate the SD of the mean.

In order to define the CD101-expressing cell subset(s) promoting protection from T1D, we assessed the distribution of myeloid and lymphoid cells in the organs of CD101-expressing and CD101-deficient NOD.B6 *Idd10* and NOD.B6 *Idd10/18* mice. The inflammatory infiltrate in the pancreata of CD101^−/−^ NOD.B6 *Idd10/18* and CD101^−/−^ NOD.B6 *Idd10* mice and respective CD101-expressing controls consisted mainly of T lymphocytes (Fig 4A) and few Gr1-expressing myeloid cells (Fig 5A and 5B). There was an enhanced proportion of CD4- over CD8-positive T lymphocytes within the TCRβ^+^ population in both CD101^−/−^ strains compared to their CD101-expressing counterparts (Fig 4B–4D). While the NOD versus B6 *Idd10* haplotypes revealed no differences in Treg percentages in the pancreatic lymph nodes (Fig 1E), Tregs were significantly reduced in the pancreatic lymph nodes (Fig 4E), but not the spleens (Fig 4F) of CD101^−/−^ NOD.B6 *Idd10* and CD101^−/−^ NOD.B6 *Idd10/18* mice compared to CD101-expressing NOD.B6 *Idd10* and NOD.B6 *Idd10/18* mice. Popliteal lymph nodes revealed also comparable Treg percentages (S8 Fig). Thus, together with the improved function of the B6 over the NOD *Cd101* allele the increased T1D frequency in CD101^−/−^ NOD.B6 *Idd10* and CD101^−/−^ NOD.B6 *Idd10/18* mice as compared to their CD101^+/+^ counterparts is associated with a reduction of Tregs in pancreatic lymph nodes suggesting that CD101 acts locally at the site of T cell priming.

### Protection from T1D correlates with an accumulation of CD101-expressing Gr1-/CD11b-double-positive myeloid cells in pancreatic tissues

We have recently reported a correlation between the CD101-dependent distribution of Gr1-positive myeloid cells and the susceptibility to T1D [31]. Thus, we evaluated the distribution of different Gr1-expressing myeloid cell populations in CD101-expressing and CD101-deficient NOD.B6 *Idd10* and NOD.B6 *Idd10/18* mice. Similar to B6 CD101^−/−^ mice [31], CD101^−/−^ NOD.B6 *Idd10* and CD101^−/−^ NOD.B6 *Idd10/18* mice have a reduction of Gr1-positive cells in the bone marrow compared to CD101-expressing congenic controls (S9A Fig). A similar tendency was also observed in the spleen (S9B Fig). To further characterize the Gr1-positive population, we used additional markers and also investigated its distribution in the pancreatic islets and pancreatic lymph nodes. As previously reported for NOD mice [4] we observed myeloid immune cells in the infiltrates of the pancreatic islets of CD101-expressing NOD.B6 *Idd10* and CD101-deficient NOD.B6 *Idd10* mice. Strikingly, however, cells infiltrating the pancreatic islets of CD101^−/−^ NOD.B6 *Idd10* mice contained substantially smaller proportions of both CD11b^+^ Gr1^−^ and CD11b^high^ Gr1^+^ cells than their CD101-expressing counterparts (Fig 5A). In contrast, the CD11b^+^ Gr1^−^ population was detected equivalently in the CD101^−/−^ and CD101^+/+^ NOD.B6 *Idd10* strains in the pancreatic lymph nodes (Fig 5B). The distribution of CD11c and F4/80 within this CD11b-positive subset in the pancreatic lymph nodes was also comparable in these two strains (S10 Fig). Similar to the islet infiltrating cells, the proportion of CD11b^high^ Gr1^+^ cells, consisting of neutrophils and myeloid-derived suppressor cells (MDSCs) [41–43], were significantly reduced in the pancreatic lymph nodes of CD101^−/−^ as compared to CD101^+/+^ NOD.B6 *Idd10* mice (Fig 5B).

### CD101 limits the expansion of diabetogenic T lymphocytes

**Fig 6:**
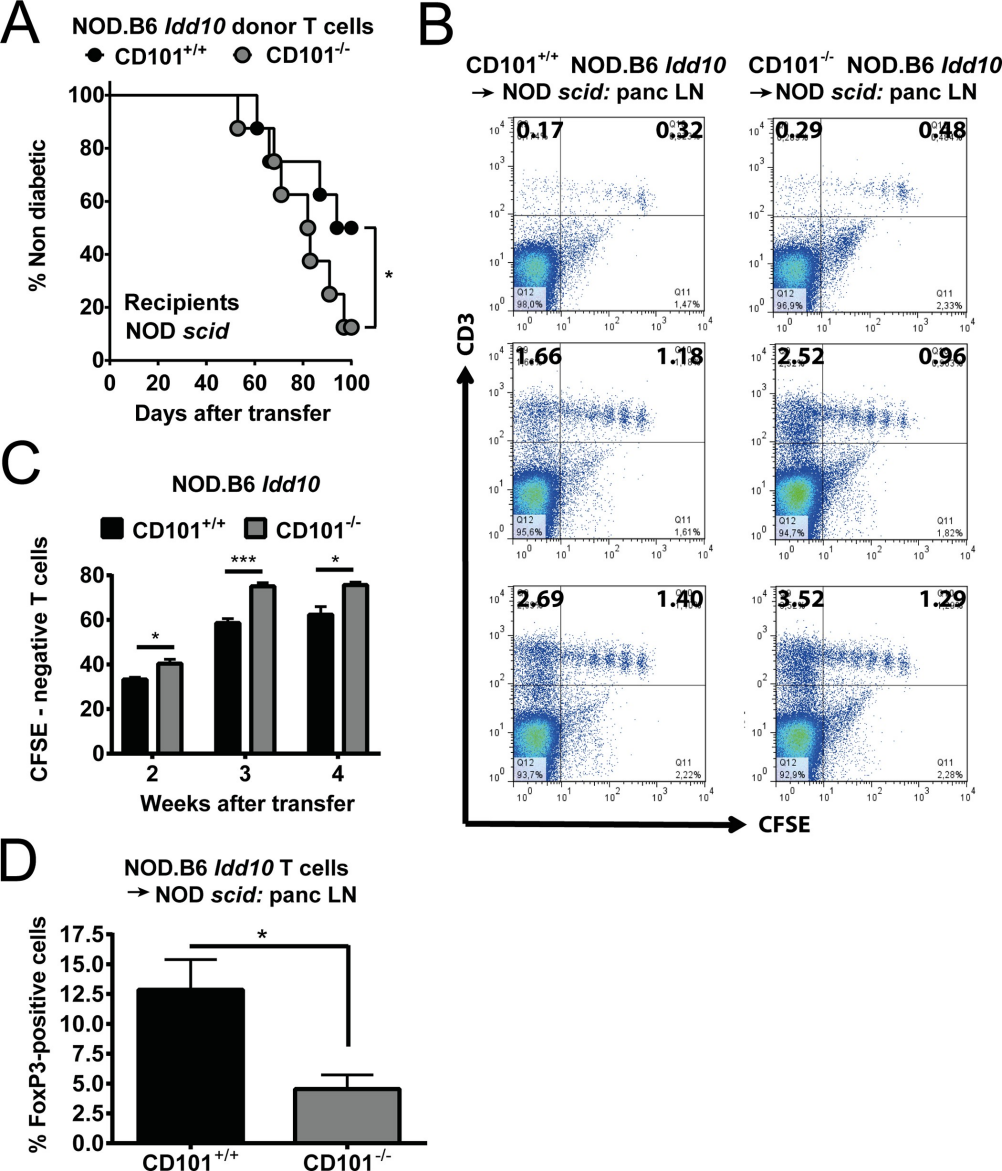
Protection from T1D is associated with a restriction of T cell expansion and an accumulation of Tregs. The frequency of T1D was assessed by the analysis of urinary glucose concentration (A) in NOD *scid* recipients upon transfer of 5x10^7^ T cells from 6-8-week-old CD101^+/+^ NOD.B6 *Idd10* or CD101^−/−^ NOD.B6 *Idd10* mice. 16 female recipients in each cohort were analyzed until day 100 after the adoptive T cell transfer. Kaplan–Meier survival curves were plotted for each mouse strain, and statistical significance was determined by log rank test (*, p<0.05). CD101 restricts the expansion of adoptively transferred T cells (B+C). T cells from CD101^+/+^ NOD.B6 *Idd10* and CD101^−/−^ NOD.B6 *Idd10* donors were CFSE-labelled and transferred into NOD *scid* recipients. 2 (upper panels), 3 (middle panels) and 4 weeks (lower panels) after transfer the division of T cells in the pancreatic lymph nodes was assessed by the analysis of CFSE dilution. The percentages of CFSE-negative cells reflecting the upper left gates in panel B were determined from all CD3-positive T lymphocytes and compared between recipient mice of CD101^+/+^ or CD101^−/−^ donors (C). Representative FACS dot plots (B) and summaries (C) of donor T cells obtained from the pancreatic lymph nodes of 3–5 NOD *scid* recipients are displayed. All cells were pre-gated on CD45. Tregs are reduced in the pancreatic lymph nodes of NOD *scid* recipients of CD101^−/−^ as compared to CD101^+/+^ NOD.B6 *Idd10* T cells (D). The percentage of FoxP3^+^ cells in total CD4 T cells are shown (n = 5 in each cohort). To assess statistical significance a one-tailed t-text was used (*, p<0.05; **, p<0.01).

To study the function of CD101 on T cells in the T1D model we purified T cells from the spleens of CD101^−/−^ and CD101-expressing NOD.B6 *Idd10* mice and transferred the CD4- and CD8-positive T cell population into lymphopenic NOD *scid* recipients. The combined transfer of CD4- and CD8-positive T lymphocytes increased T1D frequency in recipients of donor T cells originating from CD101^−/−^ NOD.B6 *Idd10* mice compared to donor T cells originating from CD101-expressing NOD.B6 *Idd10* controls (Fig 6A). The increased frequency was modest, just reaching significance (p = 0.038). The higher frequency of T1D was accompanied by a more rapid expansion of T cells from CD101^−/−^ NOD.B6 *Idd10* donors (Fig 6B and 6C). Furthermore, similar as observed in CD101^−/−^ NOD.B6 *Idd10* mice (Fig 4E), less FoxP3^+^ Tregs in relation to CD4-positive T cells accumulated in the pancreatic lymph nodes of NOD *scid* recipients from CD101^−/−^ NOD.B6 *Idd10* donors than from CD101-expressing NOD.B6 *Idd10* donors (Fig 6D). Thus, these data support the hypothesis that CD101 expression on T cells reduces effector T cell expansion, a conclusion also reached in our T cell transfer colitis studies [35].

### CD101 promotes the function of immunosuppressive Gr1-expressing myeloid cells

**Fig 7:**
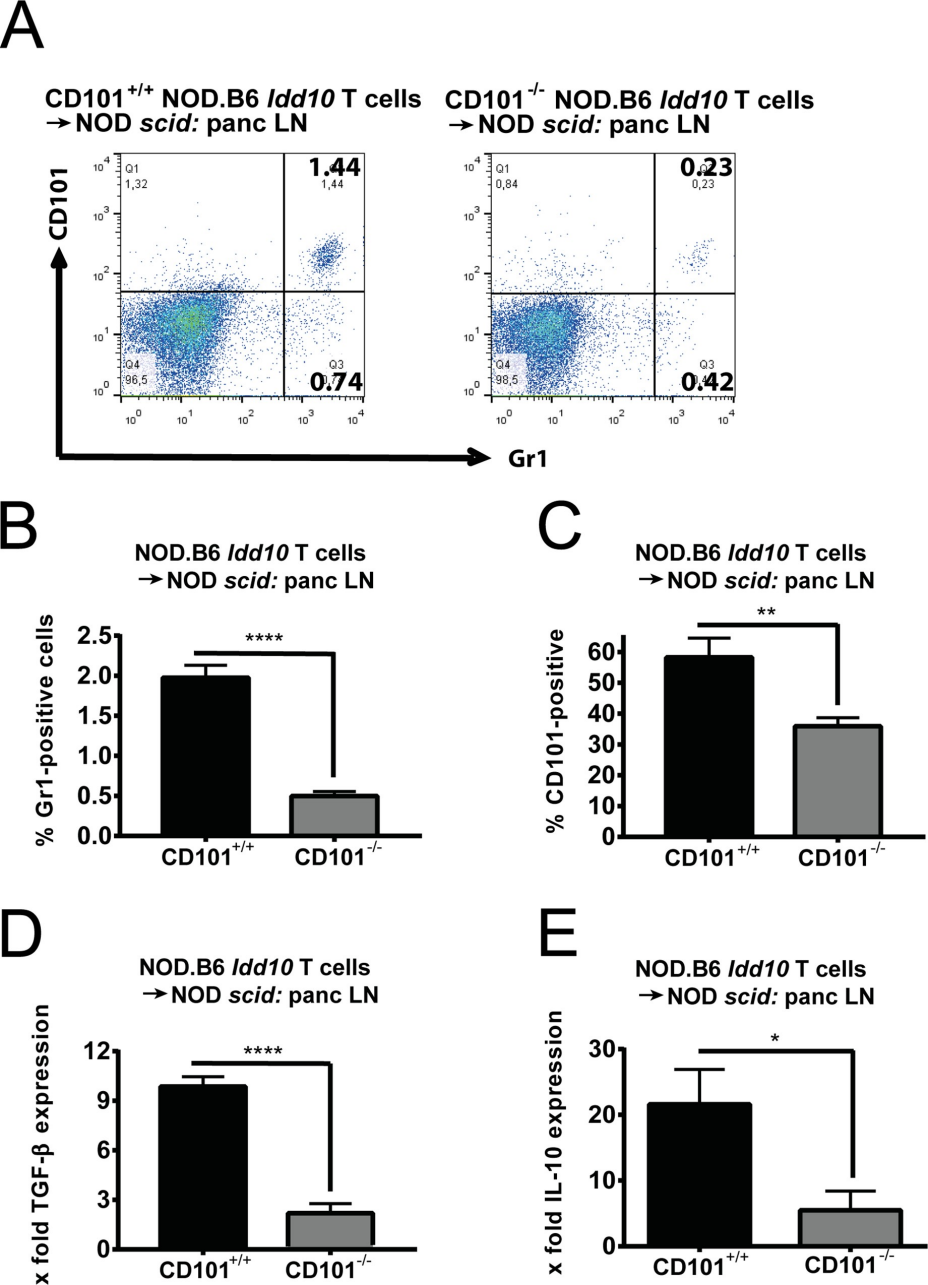
Protection from T1D is associated with an accumulation of CD101-expressing myeloid cells. Gr1-positive cells have reduced accumulation and CD101 expression (A-C) in the pancreatic lymph nodes of NOD *scid* recipients of CD101^−/−^ as compared to CD101^+/+^ NOD.B6 *Idd10* T cells. Representative FACS dot plots of Gr1-expressing myeloid cells in the pancreatic lymph nodes are displayed (A) as well as the percentages of Gr1-expressing cells (B) and the distribution of CD101 expression on them (C) compiled from 5 recipients in each cohort. Gr1-expressing myeloid cells from recipients of CD101^−/−^ donor cells express significantly less TGF-β (D) and IL-10 (E) (N = 4 in each cohort for D and E). TGF-β and IL-10 mRNA copies were determined by RT-qPCR in purified Gr1-expressing cells. The ratios of the TGF-β and IL-10 mRNA copies relative to the HPRT copies were calculated. Statistically significant differences were determined using Student´s t-tests (*, p<0.05; **, p<0.01; ***, p<0.001; ****, p<0.0001). Error bars indicate the SD of the mean.

We had observed that CD101-expressing myeloid cells decreased upon transfer of naïve CD4^+^ T cells [35]. Thus, we evaluated the composition of myeloid cells and the distribution of CD101 expression in pancreatic islets of NOD *scid* recipient mice upon combined CD4^+^/CD8^+^ T cell transfer from CD101-expressing and CD101-deficient NOD.B6 *Idd10* donors. Interestingly, significantly fewer Gr1-positive myeloid cells derived from NOD *scid* recipients accumulated in the pancreatic lymph nodes upon T cell transfer from CD101^−/−^ NOD.B6 *Idd10* donors as compared to T cells transferred from CD101^+/+^ NOD.B6 *Idd10* donors (Fig 7A and 7B). Furthermore, when CD101^−/−^ NOD.B6 *Idd10* donor T cells were transferred fewer of the accumulating Gr1-positive myeloid cells expressed CD101 (Fig 7A and 7C) and the Gr1-positive myeloid cells produced less TGF-β (Fig 7D) and IL-10 (Fig 7E) than their CD101-expressing counterparts, as observed previously for CD101-expressing myeloid cells in the gut [35].

**Fig 8:**
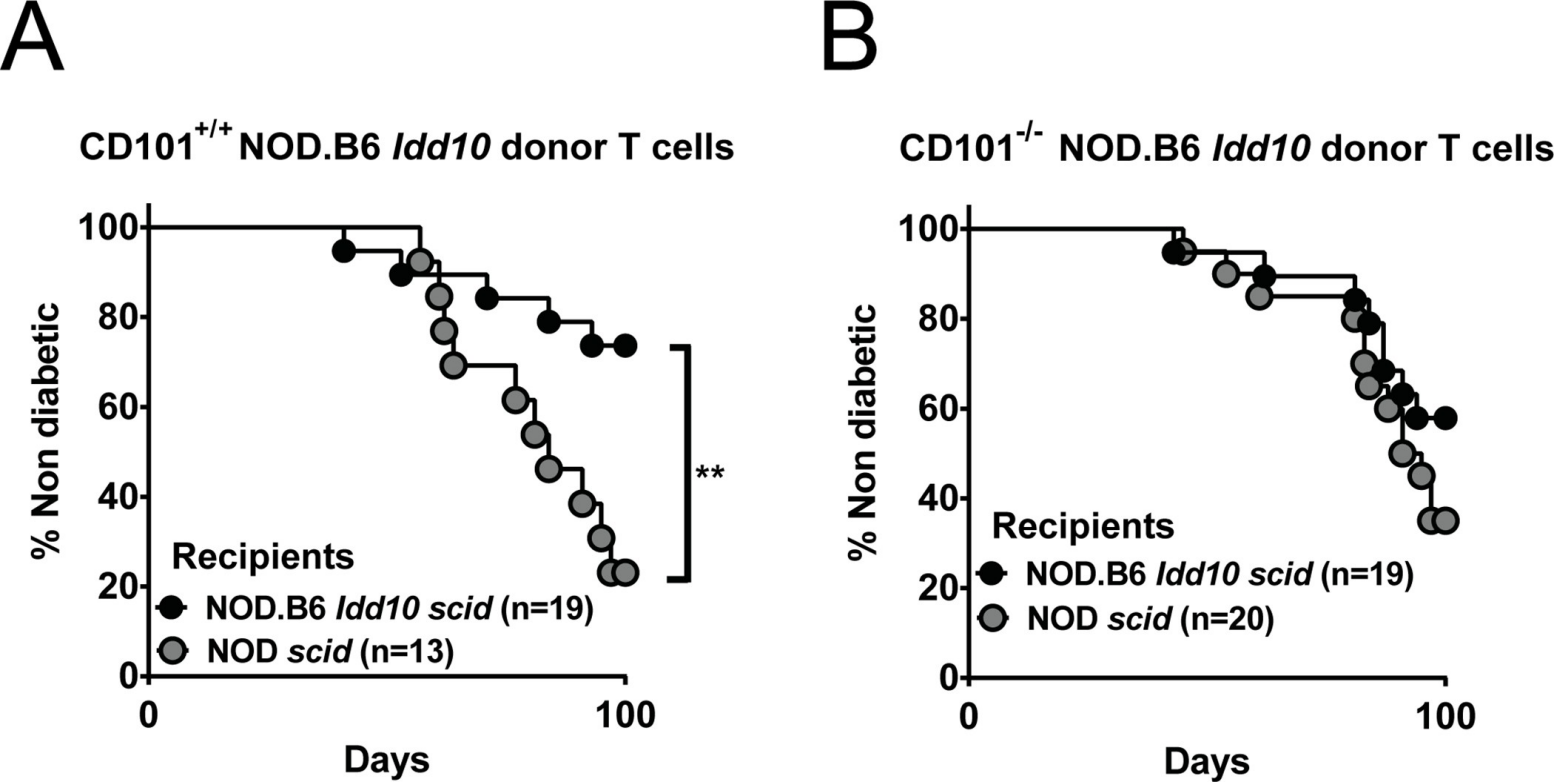
B6-derived *Idd10* region in myeloid cells as well as T cells provides the greatest degree of protection from T1D. The frequency of T1D was assessed by the analysis of urinary glucose concentration in NOD *Idd10 scid* and NOD.B6 *scid* recipients upon transfer of 5x10^7^ T cells from 6-8-week-old CD101^+/+^ NOD.B6 *Idd10* (A) or CD101^−/−^ NOD.B6 *Idd10* mice (B). The indicated numbers of female recipients in each cohort were analyzed until day 100 after the adoptive T cell transfer. Kaplan–Meier survival curves were plotted for each mouse strain, and statistical significance was determined by log rank test (*, p<0.05).

To further characterize the role of the B6 *Cd101* allele on myeloid cells for the protection from T1D, we generated NOD.B6 *Idd10 scid* mice and assessed T1D incidence in these recipients as compared to NOD *scid* recipients upon T cell transfer from CD101^+/+^ NOD.B6 *Idd10* and CD101^−/−^ NOD.B6 *Idd10* donors. The development of T1D was significantly ameliorated (p = 0.007) when T cells from CD101-expressing NOD.B6 *Idd10* donors were transferred into NOD.B6 *Idd10 scid* as compared to NOD *scid* recipients indicating that in the presence of the CD101 protein encoded by the B6 *Cd101* allele in the T cell compartment, the status of the *Cd101* allele expressed by myeloid cells determines the level of protection from T1D (Fig 8A). When CD101^−/−^ NOD.B6 *Idd10* donor T cells were used in the adoptive transfer, fewer NOD.B6 *Idd10 scid* recipients than NOD *scid* recipients developed T1D but the difference was not significant (p = 0.2; Fig 8B). In contrast to the results in Fig 6A where CD101^−/−^ NOD.B6 *Idd10* T cells mediated a modest increase in T1D upon transfer into NOD scid recipients as compared to CD101^+/+^ NOD.B6 Idd10 T cells, no difference (p = 0.35) was observed in a repeat of the same transfer combination (Fig 8A and 8B). A consideration of both results supports the conclusion that the effect of CD101 expression in T cells for influencing T1D progression is marginal when CD101 expression in the myeloid compartment is encoded by the NOD *Cd101* allele rather than the B6 *Cd101* allele. Overall our data support the hypothesis that the expression of the B6 CD101 molecule is not only important for limiting the aggressiveness of diabetogenic T cells, but also promotes the function of disease-limiting myeloid cell subsets.

## Discussion

Allelic variations within *Cd101* have been previously associated with susceptibility to T1D [31, 37]. Here, we provide further evidence that *Cd101* is *Idd10* as the genetic deletion of CD101 within the introgressed B6 *Idd10* region abolishes protection from T1D. Significantly reduced Treg frequencies in the pancreatic lymph nodes in these newly generated CD101^−/−^ NOD.B6 *Idd10* mice and an enhanced T cell expansion upon adoptive transfer accompanied the loss of protection from T1D observed in the CD101^−/−^ NOD.B6 *Idd10* strain. Furthermore, the proportion of CD101-expressing CD11b-positive myeloid cells in recipient *scid* mice was reduced upon transfer of CD101^−/−^ donor T cells correlating with a reduced IL-10 and TGF-β mRNA. Myeloid cells from CD101-expressing NOD.B6 *Idd10* mice also accumulated more frequently in pancreatic tissues than myeloid cells from CD101^−/−^ NOD.B6 *Idd10* mice. Thus, the interplay of CD101-expressing Tregs [30, 35] with CD101-positive myeloid cells appears to perpetuate an anti-inflammatory cytokine profile and limits the onset of T1D.

CD101 exhibits intrinsic effects on Treg differentiation and function and promotes the production of IL-10 by myeloid cells [35]. Here, we confirmed the pivotal role of simultaneous expression of the B6 *Cd101* allele within the myeloid cell and T lymphocyte compartment for the most complete protection from T1D. Based on similar T1D frequencies in NOD and CD101^−/−^ NOD.B6 *Idd10* mice we hypothesize that the NOD CD101 allotype present in NOD mice may not function properly due to the 10 amino acid differences from the B6 CD101 protein [37], and thus, resembles the CD101 knockout. Furthermore, the B6 *Idd10* allele enhances the expression of CD101 protein on Tregs and Gr1-expressing myeloid cells [31]. A greater understanding of the signaling and cellular interactions mediated by the CD101 protein is required to determine how the B6 and NOD *Cd101* alleles mediate, or fail to mediate, their functions.

As Gr1-expressing myeloid cells in the bone marrow are precursors for multiple myeloid lineages in the periphery, we investigated the myeloid cell composition and the distribution of myeloid surface markers in pancreatic tissues and lymph nodes in more detail. However, with the exception of CD11b, we did not detect significant differences in the expression of F4/80, Ly6C or Ly6G between Gr1-expressing myeloid cells from CD101-expressing and CD101-deficient NOD.B6 *Idd10* mice. As significantly more Gr1-positive CD11b-positive myeloid cells accumulated in CD101^+/+^ NOD.B6 *Idd10* mice than in CD101^−/−^ NOD.B6 *Idd10* mice, CD101 might promote the maturation and function of this myeloid subset. In accordance with the anti-inflammatory cytokine profile, these Gr1-expressing cells might reflect MDSCs, which have been reported to suppress T1D [44]. However, the origin of this myeloid subset and its classification into granulocytes or MDSCs need to be assessed. Alternatively, these Gr1-expressing myeloid cells might consist of a plastic MDSC and neutrophil mixture which exert an anti-inflammatory cytokine profile dependent on the expression of CD101. Alternatively, CD101 might represent a functional marker separating inflammatory neutrophils from immunoregulatory MDSCs. Further studies are needed to delineate whether alterations in the development and/or generation of additional myeloid subsets are promoted by CD101-deficiency since NOD mice have been reported to have multiple alterations in DC subsets compared to B6 mice, for example [45, 46].

Our observation that CD101 affects Gr1-expressing cells is also of clinical relevance as reduced neutrophil counts in the peripheral blood and enhanced neutrophil activity have been reported in T1D patients [47–49]. Neutrophil infiltration and neutrophil extracellular trap formation are also detected in the islets of NOD mice as early as two weeks after birth, well before the onset of overt diabetes. The blockade of neutrophil activities or neutrophil depletion at these early stages reduces the development of insulitis and diabetes in NOD mice [4]. Thus, the reduced neutrophil counts in the periphery of CD101^−/−^ NOD.B6 *Idd10* mice might be a consequence of one or more of the following reasons: *1*) impairment in the output of neutrophils from the bone marrow and/or the differentiation of neutrophils; *2*) increase in peripheral consumption/destruction; *3*) tissue sequestration. Based on our previous studies [31], we suspect that a bone marrow defect affects either the egress or the generation of myeloid cell precursors.

Decreased T1D frequency has been shown to be associated with enhanced CD101 expression on splenic Tregs of congenic NOD.B6 *Idd10* mice compared to parental NOD mice [31]. In the current study, we observed that significantly more Tregs accumulated in the pancreatic lymph nodes of CD101^+/+^ NOD.B6 *Idd10* compared to CD101^−/−^ NOD.B6 *Idd10* mice, confirming the positive effect of CD101 on Treg differentiation and function [30, 35, 50]. Thus, although NOD mice, similar to T1D patients, do not have a primary deficit in Treg percentages or numbers compared to other reference strains [17, 18, 51–53], our data imply that the B6 *Cd101* allele primarily affects Tregs within the T cell compartment. These animal studies are also in line with a recent report on distinct T1D patient cohorts raising the possibility of *CD101* being a susceptibility gene for human T1D [36]. In addition, since IL-2 administration acts via pancreatic Tregs [54] and CD101 sensitizes Tregs to IL-2 signals [35], the accumulation of CD101^+^ Tregs in NOD mice carrying the B6 *Cd101* allele likely contributes to the protection from T1D.

CD101 expression restrains the accumulation and expansion of diabetogenic CD4- and CD8-positive T lymphocytes and reduces T1D frequency in lymphopenic recipient mice upon mixed CD4/CD8 T cell transfers from CD101^+/+^ NOD.B6 *Idd10* mice. In particular, CD8-positive T cells might be interesting to study further since recent reports claimed altered functions between CD101-expressing CD8-positive T lymphocytes and their CD101-negative counterparts [55, 56]. Thus, in summary, our data clearly indicate that CD101 promotes the accumulation of anti-inflammatory lymphoid and myeloid cells and slows or halts disease in an autoimmune-prone background when sufficiently expressed.

## Methods

### Ethics statement

The experiments were conducted according to the Institutional Animal Care and Use Committee guidelines of the Cincinnati Children’s Hospital (IACUC protocol number Protocol 8D02011) and approved by the Animal Welfare Committee of the local government (Regierung von Mittelfranken, Ansbach, Germany; protocol: 54–2532.1-30/10). Daily inspections were performed to minimize animal suffering. Mice with signs of discomfort or disease were euthanized immediately by C02 and cervical dislocation.

### Mice

NOD/MrkTac (NOD) and NOD *scid* mice were obtained from Taconic Farms (Germantown, NY, USA). The development of the NOD.B6 *Idd10* (N16) (Taconic line 3538) and NOD.B6 *Idd10/18* (N10) (Taconic line 7754) strains were described previously [31, 39]. NOD.B6 *Idd10* and NOD *scid* mice were intercrossed and F2 mice homozygous for the *Idd10* and *scid*-containing regions selectively bred to develop the NOD.B6 *Idd10 scid* strain. B6 CD101^−/−^ mice [31] were backcrossed onto the NOD background to develop the CD101^−/−^ NOD.B6 *Idd10* (N11) and CD101^−/−^ NOD.B6 *Idd10/18* strains (N10). Polymorphic markers near and within the *Idd10* and *Idd18* regions were used to define recombination events as similar as possible to the boundaries of these regions (S1 Table) as previously defined. The CD101^−/−^ NOD.B6 *Idd10* and CD101^−/−^ NOD.B6 *Idd10/18* strains were free of B6-derived genetic segments outside of the selected areas as defined by screening with a 1449 polymorphic marker panel as described previously [31]. All mice were raised and kept in a specific pathogen-free environment and used at 3–15 weeks of age for cellular and molecular analyses and for T1D frequency studies until the age of 200 days. The appropriate institutional review committee approved the T1D frequency studies performed at Taconic Farms.

### Preparation of pancreatic islets

Mice were euthanized and pancreata were perfused with a 1.5 mg/mL solution of collagenase P (Roche Molecular Biochemicals, Mannheim, Germany), dissected from surrounding tissues and cut into small pieces. The digestion buffer was supplemented with 1 mM PMSF, 100 μM leupeptin and 1 μM pepstatin A (Sigma-Aldrich, Taufkirchen, Germany). Pancreata were digested at 37°C for 10 min in a shaking water bath. The digestion was stopped by adding HBSS containing 5% FCS. The tissue suspension was washed three times and centrifuged through a discontinuous Ficoll gradient (23, 20.5 and 11%; Sigma-Aldrich, Taufkirchen, Germany) at room temperature. The purified islets were disrupted by adding 1 mL of cell dissociation buffer (GIBCO/Thermo Fisher Scientific, Waltham, MA, USA) for 10 minutes at 37°C. The obtained cells were washed, resuspended and used for analyses.

### Adoptive T cell transfer experiments

5x10^7^ spleen cells enriched in TCRβ-positive T cells (consisting of about 2/3 CD4-positive T cells and of about 1/3 CD8-positive T cells) from 6-8-week-old donor female mice were transferred intraperitoneally into 6-8-week-old NOD *scid* or NOD.B6 *Idd10 scid* recipients. B cells, NK cells, DCs, granulocytes or macrophages were depleted using Auto-MACS (Miltenyi Biotec, Bergisch Gladbach, Germany) and PE- or APC-fluorescence coupled beads against CD19, NKp46, Gr1, CD11c or F4/80 from NOD mice before transfer following the manufacturer's instructions with purity control by FACS.

For cell division studies donor T cells were labeled with 5 μM CFSE prior to transfer according to the manufacturer's instructions (Molecular Probes/Thermo Fisher Scientific, Waltham, MA, USA and BD Pharmingen/BD Biosciences, San Diego, CA, USA).

### Flow cytometry, intracellular cytokine staining and cell purification

Single-cell suspensions were prepared from the spleen, lymph nodes, pancreas and bone marrow. Red blood cells were not removed. Cell-surface expression of CD45.1 (clone A20), CD45.2 (clone 104), CD11c (clone N418), CD11b (clone M1/70), Ly6C (clone HK1.4), Ly6G/Gr1 (clone RB6-8C5), F4/80 (clone BM8), B220 (clone RA3-6B2), the β-chain of the TCR (clone H57-597), CD3 (clones 145-2C11 and 17A2), CD4 (clone GK1.5) and CD8a (clone 53–6.7) was detected using fluorescently labeled mAbs obtained from eBioscience (San Diego, CA, USA). PE-labeled anti-CD101 (clone 307707) was obtained from R&D Systems. Intracellular Foxp3 was detected with a staining kit following the manufacturer´s instructions (eBioscience). Cells were analyzed on an LSR II or a BD FACS Canto II (BD Biosciences, San Diego, CA, USA) with FlowJo software (Tree Star, Ashland, OR, USA). Gr1-expressing cells were purified on a FACS Aria II (BD Biosciences, Franklin Lakes, NJ, USA) (purity of > 98%).

### Diabetes frequency studies

All diabetes cumulative frequency studies were conducted using female mice. The presence of T1D was tested every 14 d beginning at 84 d of age by the detection of urinary glucose >500 mg/dl using Diastix (Miles, Elkhart, IN, USA). Overt diabetes was confirmed by a blood sugar level of >200 mg/dl. Studies were terminated at 196 d of age. Kaplan–Meier survival curves were plotted for each mouse strain, and these were compared using the log rank test (Prism4 software; GraphPad).

### Histology and H&E staining

Pancreatic tissue was fixed in 10% buffered formalin, embedded in paraffin, and cut into 5 μm thick sections. Pancreas sections were deparaffinized, stained with H&E by the Department of Pathology and the Medical Department I of the FAU Erlangen-Nürnberg, and evaluated microscopically in a double-blinded manner. H&E–stained sections were scored for insulitis. At least 10 islets per mouse present on two or three non-adjacent pancreas sections were scored as either 0, no infiltration; 1, peri-insulitis; 2, mild-invasive insulitis; or 3, severe invasive insulitis. The average score of each pancreas was calculated and used for statistical analysis.

### cDNA synthesis and real-time qPCR for mRNA expression analysis

cDNA was synthesized using a High-capacity cDNA Reverse Transcription Kit (Applied Biosystems/Thermo Fisher Scientific, Waltham, MA, USA) following the manufacturer’s instructions. Quantitative (q) -PCRs were performed as described [33] using specific primers (Thermo Scientific) and pre-designed probes (Roche, Basel, Switzerland): TGF-β (forward: 5´- gtggtgtccccacacagg-3´; reverse: 5´-ccagggctgtaaccacttg-3´) and IL-10 (forward: 5´- cagagccacatgctcctaga-3´; reverse: 5´-gtccagctggtcctttgttt-3´). Gene expression was calculated relative to the house keeping gene HPRT (forward: 5´-tcctcctcagaccgctttt-3´; reverse: 5´-cctggttcatcatcgctaatc-3´ or Applied Biosystems assay Mm00446968_m1) using the ΔΔCt algorithm.

### Statistical analysis

Samples were analyzed for normal distribution by a Kolmogorov-Smirnov test. According to the results, statistical significance in normal distributed samples were analyzed by one-way ANOVA with posthoc test (Bonferroni) and Student’s t-test, and samples failing the normal distribution test by Kruscal-Wallis Test with posthoc (Dunn’s multiple comparison) or Mann-Whitney U test as indicated in the respective experiments. A sample size of at least three (n = 3) was used for each sample group in a given experiment, and a p value of 5% (*; p 0.05), 1% (**; p 0.01), 0.1% (***; p 0.001) or 0.01% (****; p 0.0001) was considered significant to accept the alternate hypothesis. GraphPad Prism software was used for statistical analysis.

## Supporting information

S1 Fig CD101 is primarily expressed on CD11b^+^ myeloid and CD3^+^ T cells. The cell composition and the distribution of CD101 expression in the indicated organs of NOD and NOD.B6 *Idd10* mice were determined by flow cytometry. All cells were pre-gated on CD45. Representative FACS plots are shown for CD101 versus CD11b, following the gating out of TCRβ-positive T cells (A). The percentage of CD101-expressing CD11b-positive myeloid cells (B) and the distribution of myeloid (CD11b^+^) cells and T lymphocytes (CD3^+^) among CD101-expressing, CD45-positive cells (C) in the indicated organs was compiled from 5 and 3 individual NOD.B6 *Idd10* mice, respectively. Error bars indicate the SD of the mean.
(TIF)

S2 Fig CD101-positive T cells accumulate in the pancreatic lymph nodes of NOD.B6 *Idd10* mice. The expression of CD101 and FoxP3 on TCRβ-positive T cells was assessed by flow cytometry in the spleens, pancreatic and popliteal lymph nodes of 4-week-old CD101^+/+^ NOD.B6 *Idd10* mice and CD101^-/-^ NOD.B6 *Idd10* mice. Representative staining panels for each mouse strain and organ are displayed.
(TIF)

S3 Fig CD101-positive T cells accumulate in the pancreatic lymph nodes of NOD.B6 Idd10 mice. Representative FACS dot plots for the expression of CD101 on T cells from NOD and NOD.B6 *Idd10* mice in inguinal lymph nodes (A) as well as the summaries for the number of CD101-expressing T cells (B) and the mean fluorescence intensities for CD101 (C) in 5 individual mice per group are displayed. Comparisons between groups were performed using the Mann–Whitney nonparametric test (*, p<0.05; **, p<0.01). Error bars indicate the SD of the mean.
(TIF)

S4 Fig Tregs in the popliteal lymph nodes of NOD and NOD.B6 *Idd10* mice are similarly distributed. The percentage of CD101-expressing Tregs (A) as well as the percentage of FoxP3-positive Tregs (B) was compiled from the popliteal lymph nodes of 4 individual mice at 4, 8, 12 and 14 weeks. Comparisons between groups at the indicated time points were performed using the Mann–Whitney nonparametric test. Error bars indicate the SD of the mean.
(TIF)

S5 Fig Myeloid cell and lymphocyte subsets in NOD and NOD.B6 *Idd10* mice are similarly distributed. The genotype-dependent expression of CD11c^+^ (A), Gr1^+^ (B), F4/80^+^ (C) or TCRβ^+^ CD44^+^ (D) from the spleens and pancreatic lymph nodes of 6 individual NOD and NOD.B6 *Idd10* mice are summarized. Groups of mice were compared by non-parametric Mann-Whitney tests. Error bars indicate the SD of the mean.
(TIF)

S6 Fig CD101-expression protects NOD.B6 *Idd10* mice from T1D. The frequency of T1D was assessed by the analysis of urinary glucose concentration in the indicated number of female CD101^+/+^ NOD.B6 *Idd10*, CD101^+/−^ NOD.B6 *Idd10* and CD101^−/−^ NOD.B6 *Idd10* mice bred from CD101^+/−^ NOD.B6 *Idd10* heterozygous breeders. Data from the CD101^+/+^ NOD.B6 *Idd10* and CD101^+/−^ NOD.B6 *Idd10* progeny are also shown in Fig 3B. For T1D frequency comparisons Kaplan–Meier survival curves were plotted for each mouse strain, and statistical significance was determined by log rank test (**, p<0.01; ns, not significant).
(TIF)

S7 Fig CD101-expression protects NOD.B6 *Idd10* and NOD.B6 *Idd10/18* mice from insulitis. Representative pictures of H&E-stained pancreas sections from 10-week-old CD101^−/−^ NOD.B6 *Idd10* and CD101^+/+^ NOD.B6 *Idd10* female mice (A) as well as for individual insulitis scores (B) are displayed.
(TIF)

S8 Fig Tregs in the popliteal lymph nodes of CD101^+/+^ and CD101^-/-^ NOD.B6 *Idd10* mice are similarly distributed. The percentage of FoxP3-positive Tregs was compiled from the popliteal lymph nodes of five individual mice per time point. Comparisons between groups were performed using Student´s t-tests. Error bars indicate the SD of the mean.
(TIF)

S9 Fig CD101-expression promotes the expansion of Gr1-positive myeloid cells in NOD.B6 *Idd10* and NOD.B6 *Idd10/18* mice. The cell composition in the bone marrow (A) and the spleens (B) of the indicated mouse strains was assessed by flow cytometry. Data for the percentage of Gr1^+^ cells from 17 individual female mice at the age of 10–15 weeks are compiled from four independent experiments. Statistical differences were determined using Mann-Whitney tests (*, p<0.05; ***, p<0.001). Error bars indicate the SD of the mean.
(TIF)

S10 Fig The expression of F4/80 and CD11c is similar within the CD11b-positive Gr1-negative subset in CD101^+/+^ and CD101^-/-^ NOD.B6 *Idd10* mice. The percentages for the distribution of F4/80- (A) and CD11c- (B) expression within the CD11b-positive Gr1-negative myeloid cell subset in the pancreatic lymph nodes of four individual CD101^+/+^ NOD.B6 *Idd10* and CD101^-/-^ NOD.B6 *Idd10* mice are displayed. Comparisons between groups were performed using Student´s t-tests. Error bars indicate the SD of the mean.
(TIF)

S1 Table Identification of NOD.B6 congenic mice used in this study. The table contains the markers used to define the mouse lines in this study. The marker name, GRC38 coordinate and primer sequences are provided, as well as the method for assessing the polymorphism.
(DOCX)
