# Supplementary figures and images for: Genetic and functional data identifying Cd101 as a type 1 diabetes (T1D) susceptibility gene in nonobese diabetic (NOD) mice
Source: PLoS Genet. 2019 Jun 14;15(6):e1008178. doi: 10.1371/journal.pgen.1008178 (PMC6568395; doi:10.1371/journal.pgen.1008178)

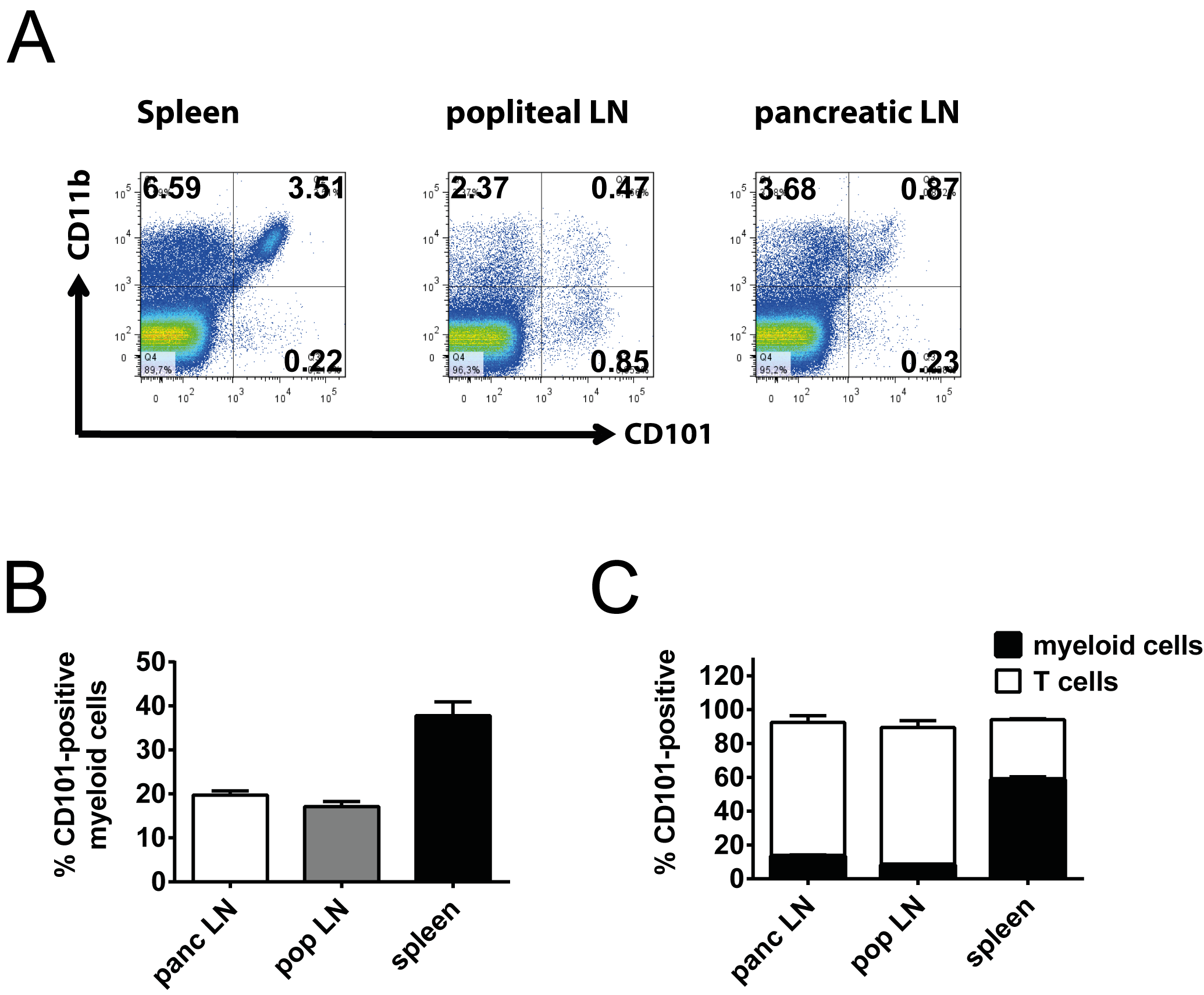

Supplement: S1 Fig — The cell composition and the distribution of CD101 expression in the indicated organs of NOD and NOD.B6 Idd10 mice were determined by flow cytometry. All cells were pre-gated on CD45. Representative FACS plots are shown for CD101 versus CD11b, following the gating out of TCRβ-positive T cells (A). The percentage of CD101-expressing CD11b-positive myeloid cells (B) and the distribution of myeloid (CD11b+) cells and T lymphocytes (CD3+) among CD101-expressing, CD45-positive cells (C) in the indicated organs was compiled from 5 and 3 individual NOD.B6 Idd10 mice, respectively. Error bars indicate the SD of the mean. (TIF) [file pgen.1008178.s001.tif]

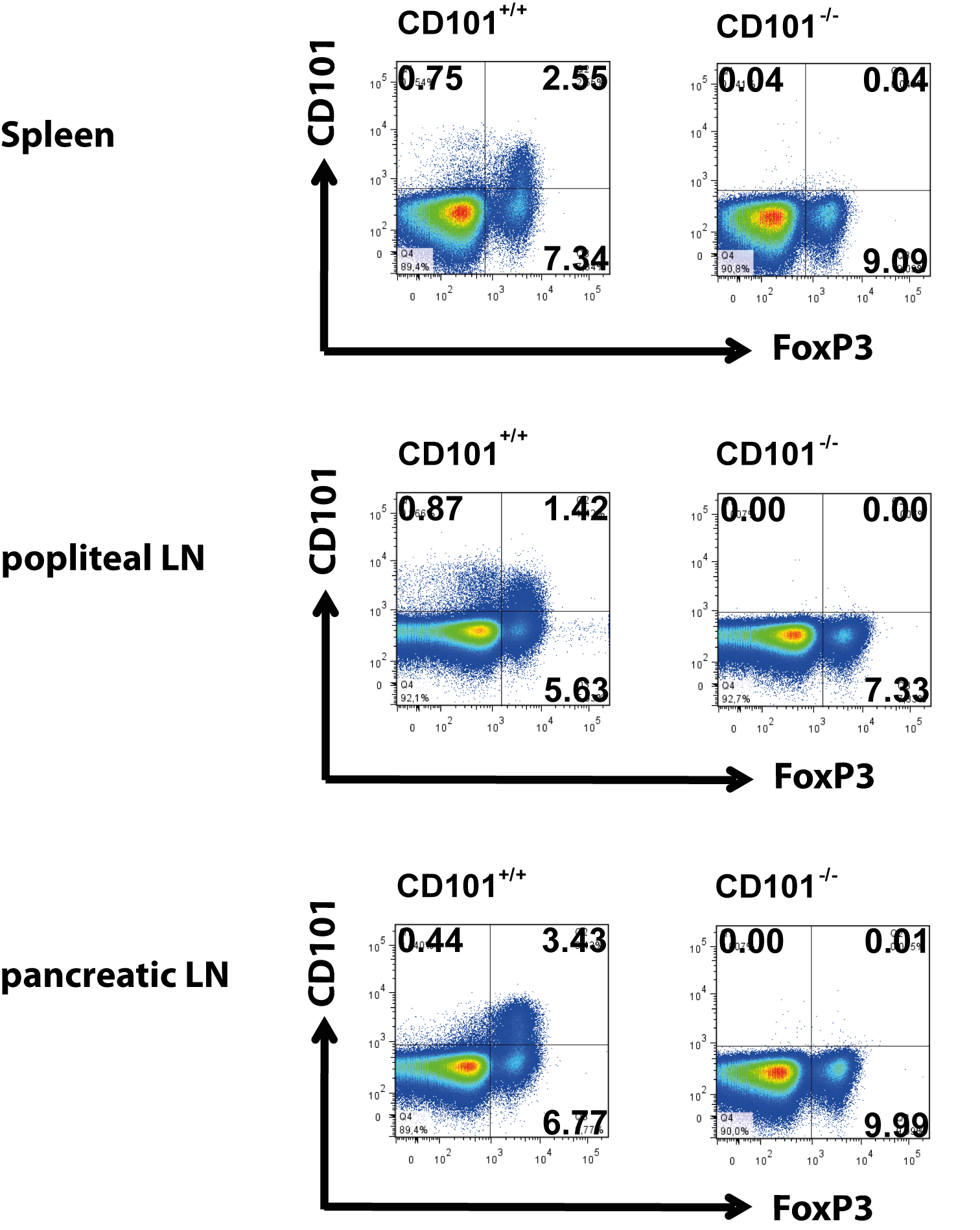

Supplement: S2 Fig — The expression of CD101 and FoxP3 on TCRβ-positive T cells was assessed by flow cytometry in the spleens, pancreatic and popliteal lymph nodes of 4-week-old CD101+/+ NOD.B6 Idd10 mice and CD101-/- NOD.B6 Idd10 mice. Representative staining panels for each mouse strain and organ are displayed. (TIF) [file pgen.1008178.s002.tif]

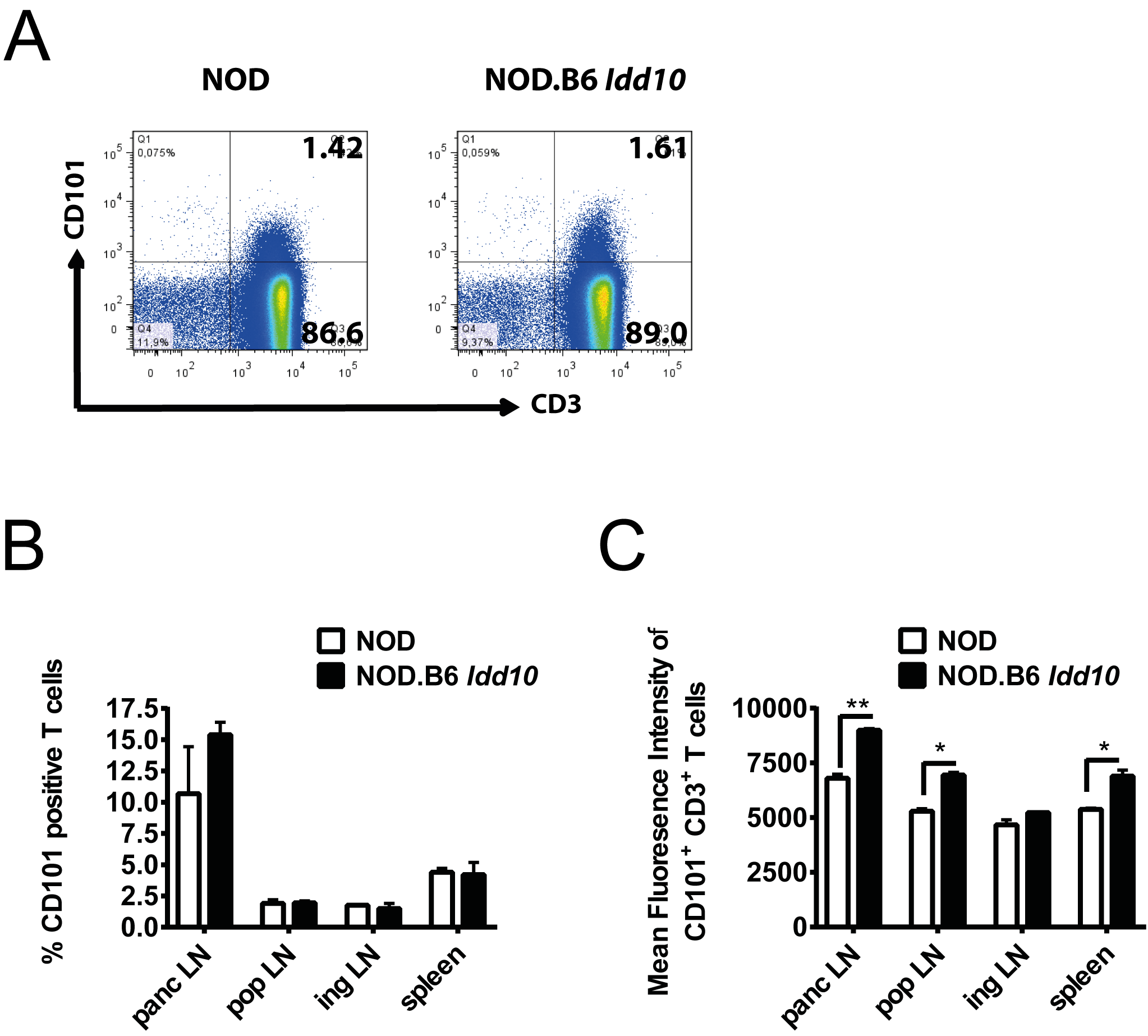

Supplement: S3 Fig — Representative FACS dot plots for the expression of CD101 on T cells from NOD and NOD.B6 Idd10 mice in inguinal lymph nodes (A) as well as the summaries for the number of CD101-expressing T cells (B) and the mean fluorescence intensities for CD101 (C) in 5 individual mice per group are displayed. Comparisons between groups were performed using the Mann–Whitney nonparametric test (*, p<0.05; **, p<0.01). Error bars indicate the SD of the mean. (TIF) [file pgen.1008178.s003.tif]

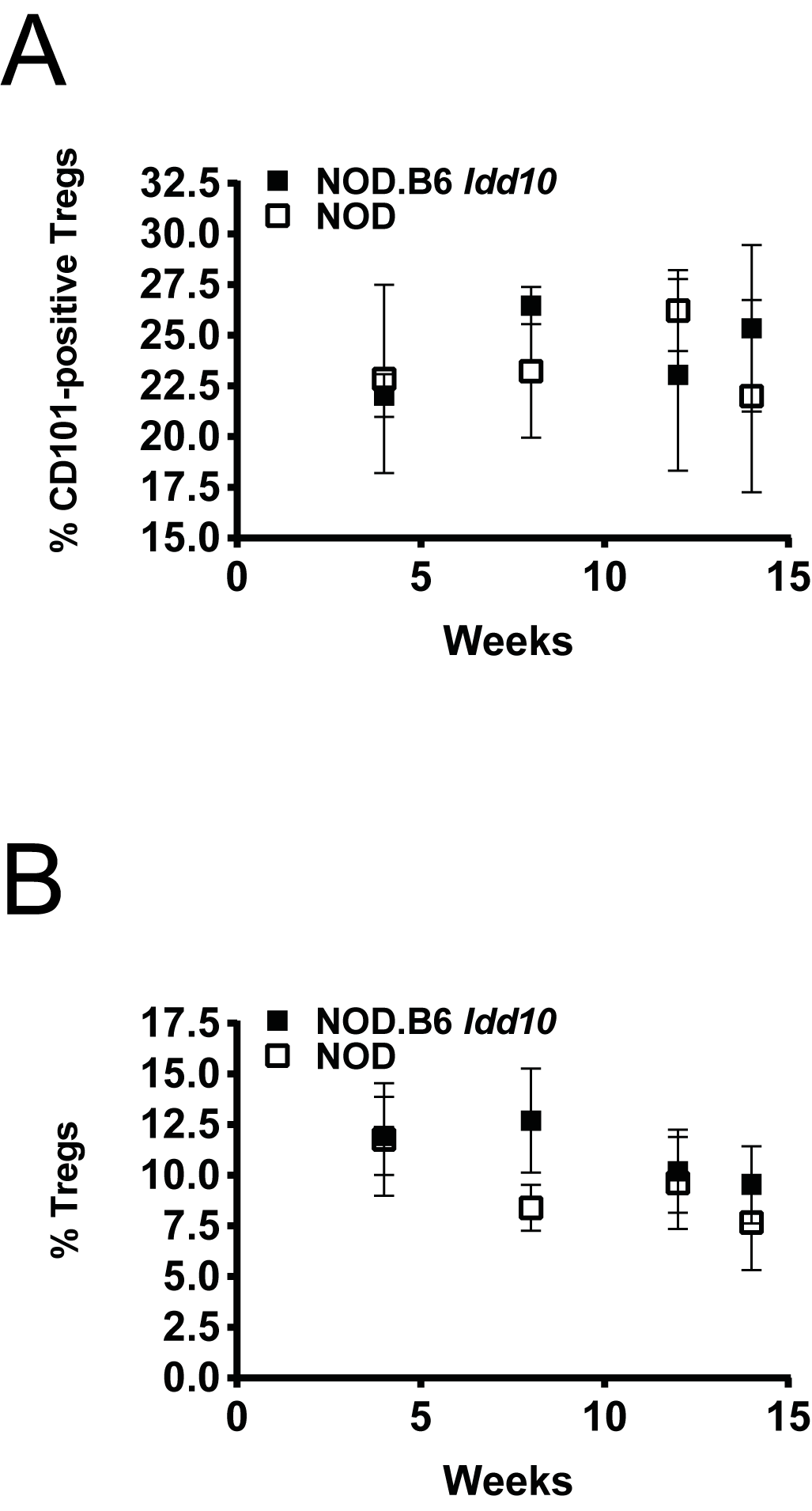

Supplement: S4 Fig — The percentage of CD101-expressing Tregs (A) as well as the percentage of FoxP3-positive Tregs (B) was compiled from the popliteal lymph nodes of 4 individual mice at 4, 8, 12 and 14 weeks. Comparisons between groups at the indicated time points were performed using the Mann–Whitney nonparametric test. Error bars indicate the SD of the mean. (TIF) [file pgen.1008178.s004.tif]

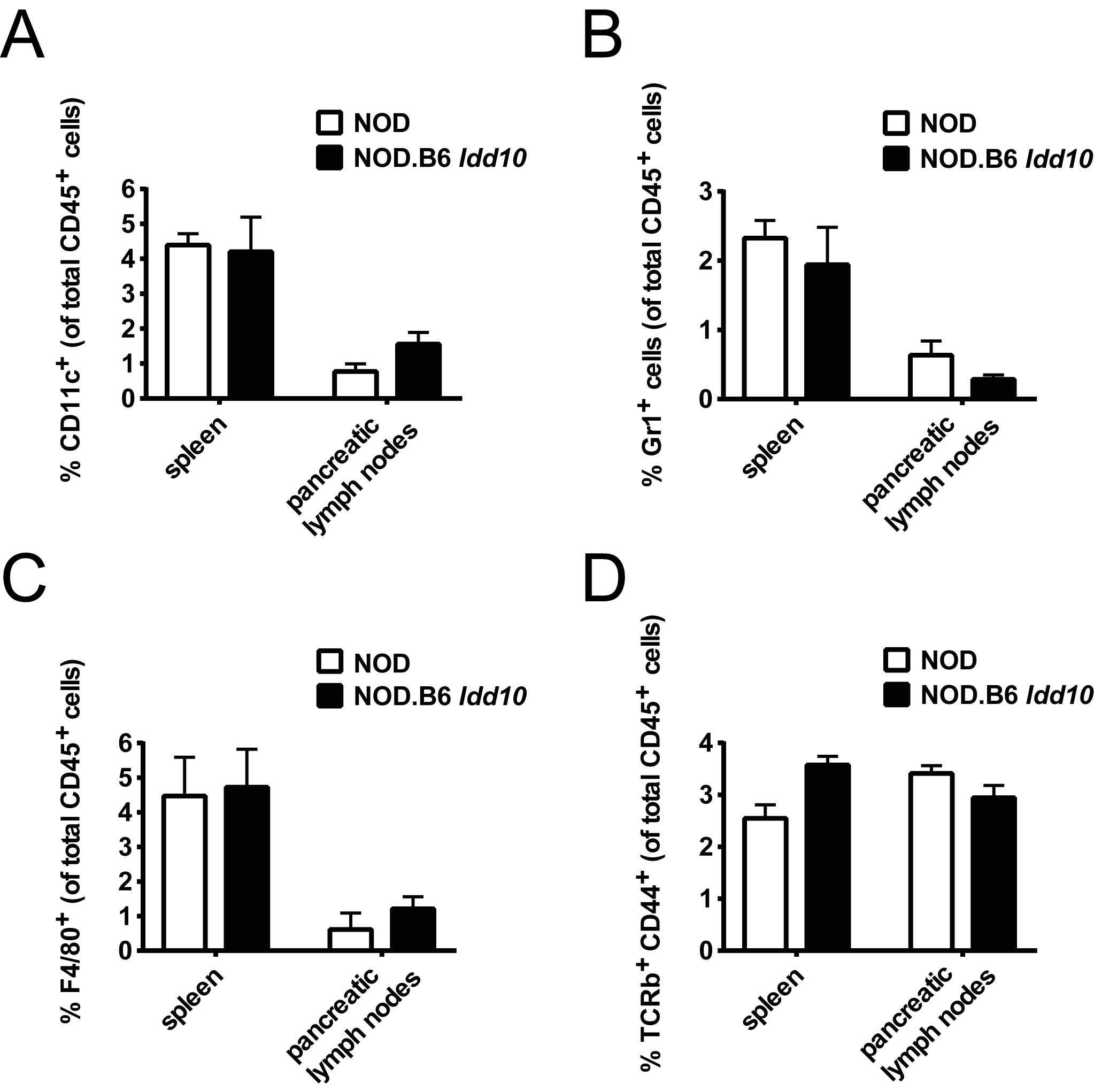

Supplement: S5 Fig — The genotype-dependent expression of CD11c+ (A), Gr1+ (B), F4/80+ (C) or TCRβ+ CD44+ (D) from the spleens and pancreatic lymph nodes of 6 individual NOD and NOD.B6 Idd10 mice are summarized. Groups of mice were compared by non-parametric Mann-Whitney tests. Error bars indicate the SD of the mean. (TIF) [file pgen.1008178.s005.tif]

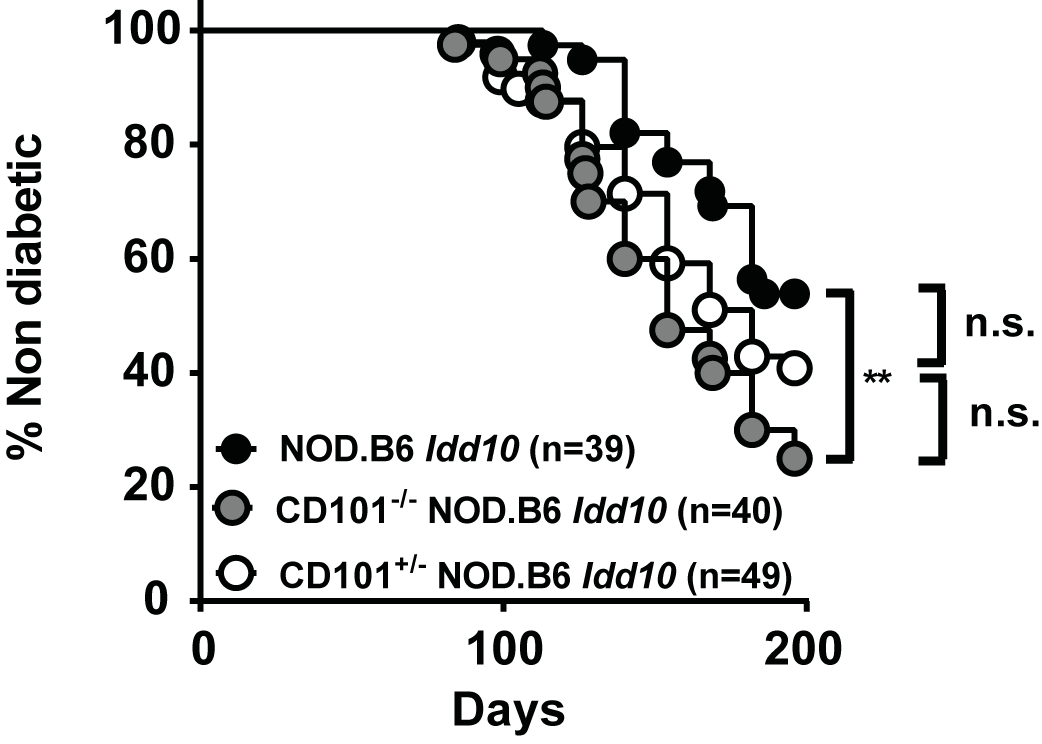

Supplement: S6 Fig — The frequency of T1D was assessed by the analysis of urinary glucose concentration in the indicated number of female CD101+/+ NOD.B6 Idd10, CD101+/− NOD.B6 Idd10 and CD101−/− NOD.B6 Idd10 mice bred from CD101+/− NOD.B6 Idd10 heterozygous breeders. Data from the CD101+/+ NOD.B6 Idd10 and CD101+/− NOD.B6 Idd10 progeny are also shown in Fig 3B. For T1D frequency comparisons Kaplan–Meier survival curves were plotted for each mouse strain, and statistical significance was determined by log rank test (**, p<0.01; ns, not significant). (TIF) [file pgen.1008178.s006.tif]

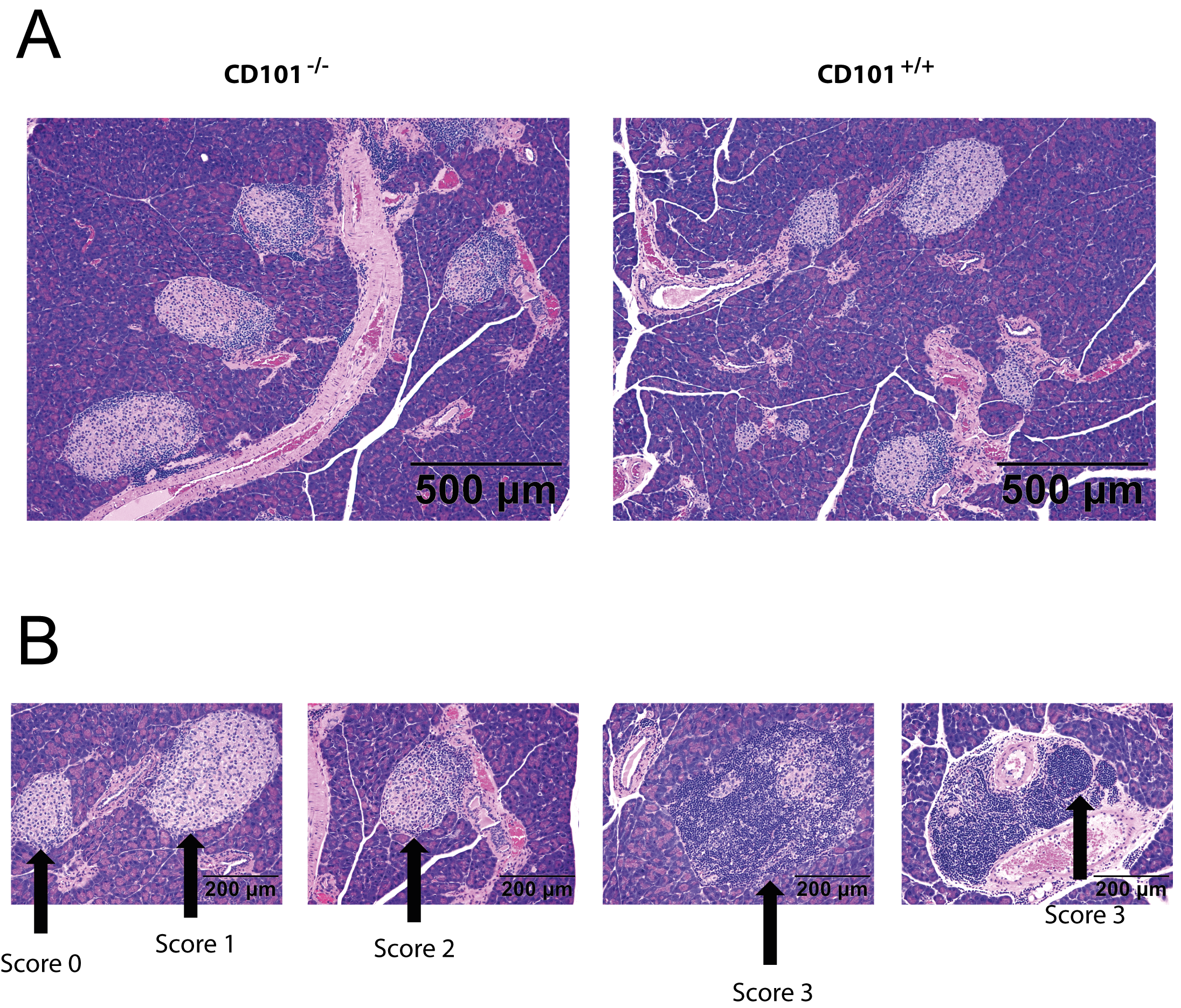

Supplement: S7 Fig — Representative pictures of H&E-stained pancreas sections from 10-week-old CD101−/− NOD.B6 Idd10 and CD101+/+ NOD.B6 Idd10 female mice (A) as well as for individual insulitis scores (B) are displayed. (TIF) [file pgen.1008178.s007.tif]

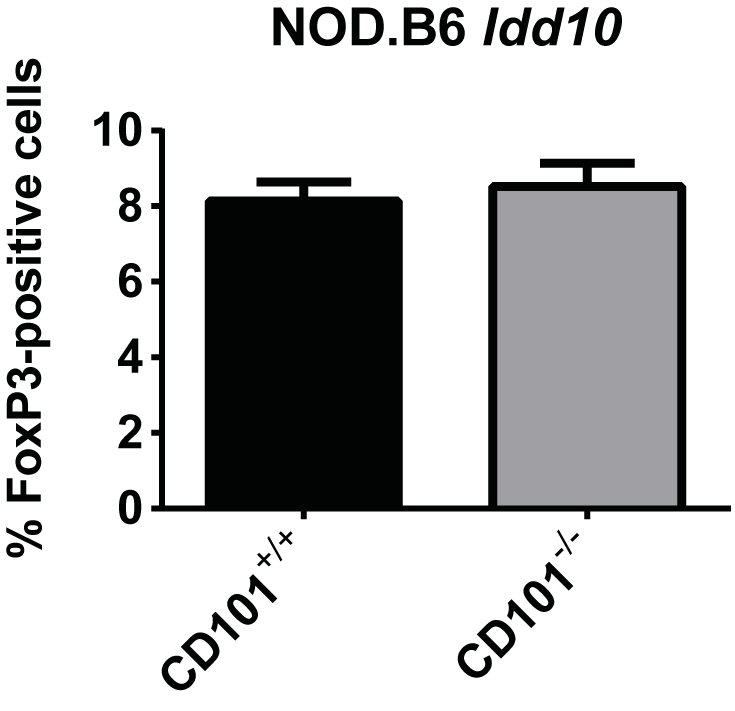

Supplement: S8 Fig — The percentage of FoxP3-positive Tregs was compiled from the popliteal lymph nodes of five individual mice per time point. Comparisons between groups were performed using Student´s t-tests. Error bars indicate the SD of the mean. (TIF) [file pgen.1008178.s008.tif]

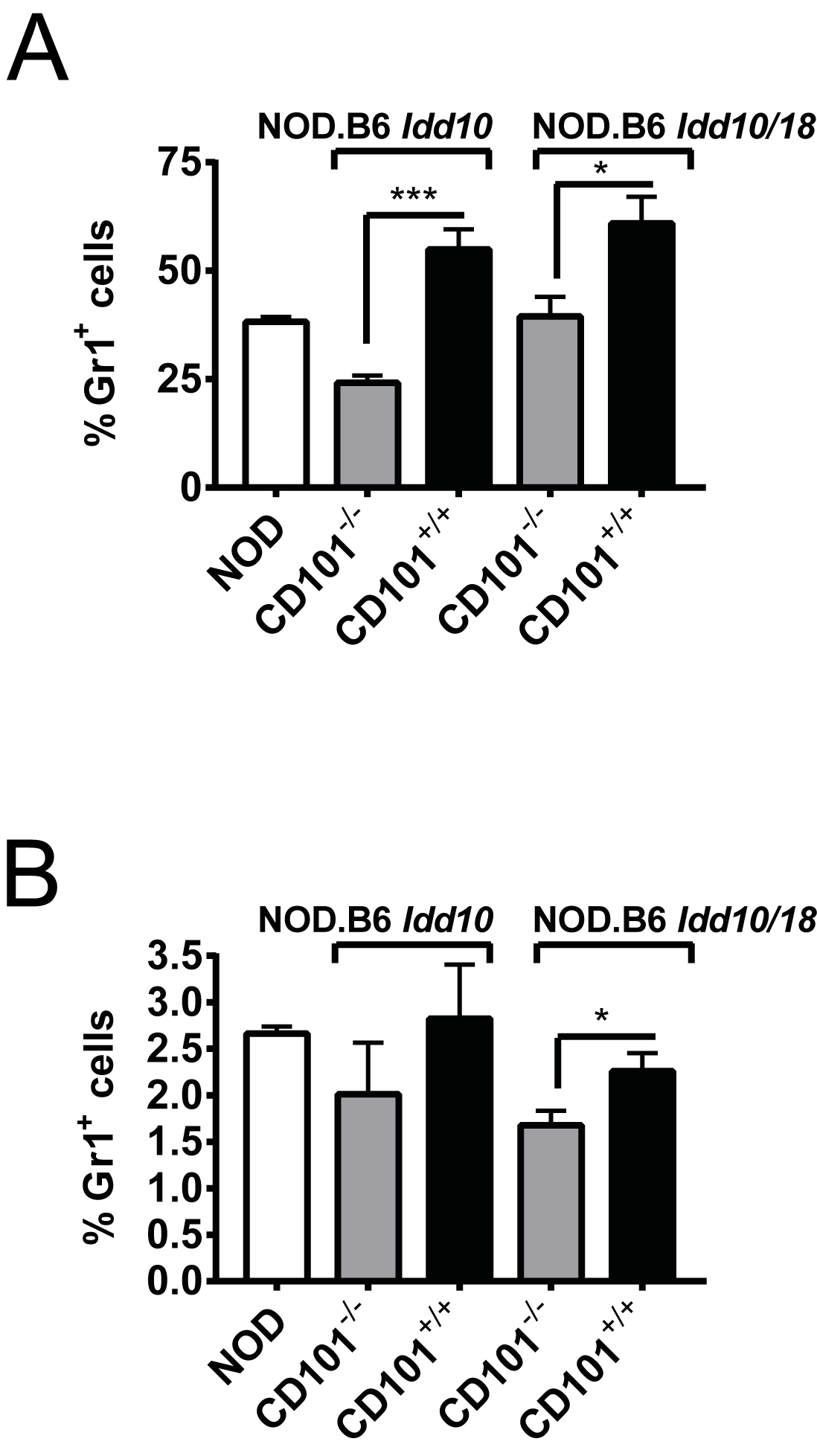

Supplement: S9 Fig — The cell composition in the bone marrow (A) and the spleens (B) of the indicated mouse strains was assessed by flow cytometry. Data for the percentage of Gr1+ cells from 17 individual female mice at the age of 10–15 weeks are compiled from four independent experiments. Statistical differences were determined using Mann-Whitney tests (*, p<0.05; ***, p<0.001). Error bars indicate the SD of the mean. (TIF) [file pgen.1008178.s009.tif]

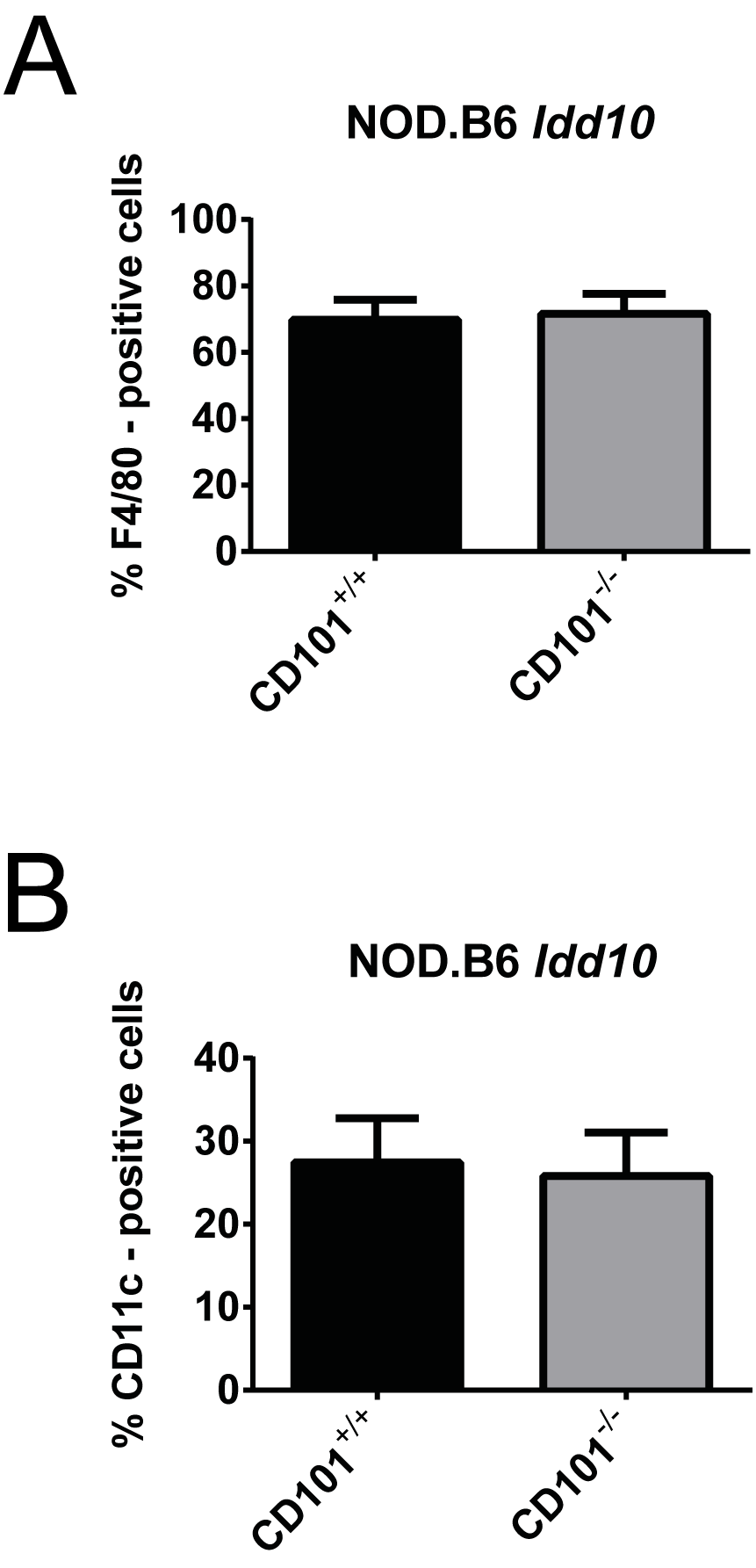

Supplement: S10 Fig — The percentages for the distribution of F4/80- (A) and CD11c- (B) expression within the CD11b-positive Gr1-negative myeloid cell subset in the pancreatic lymph nodes of four individual CD101+/+ NOD.B6 Idd10 and CD101-/- NOD.B6 Idd10 mice are displayed. Comparisons between groups were performed using Student´s t-tests. Error bars indicate the SD of the mean. (TIF) [file pgen.1008178.s010.tif]
