# Supplementary material for: Genetic and functional data identifying Cd101 as a type 1 diabetes (T1D) susceptibility gene in nonobese diabetic (NOD) mice
Source: PLoS Genet. 2019 Jun 14;15(6):e1008178. doi: 10.1371/journal.pgen.1008178 (PMC6568395; doi:10.1371/journal.pgen.1008178)
Supplement: S1 Table — The table contains the markers used to define the mouse lines in this study. The marker name, GRC38 coordinate and primer sequences are provided, as well as the method for assessing the polymorphism. (DOCX) [file pgen.1008178.s011.docx]

| Supplemental table 1: The markers used to define the mouse lines used in this study. The marker name, GRC38 coordinate and primer sequences are provided, as well as the method for assessing the polymorphism. | | | | |
| --- | --- | --- | --- | --- |
|  |  |  |  |  |
| **Marker** | **Coordinates** | **Forward** | **Reverse** | **Method** |
| D3Mit157 | 3:97279136-97279285 | ACATGTGGCACATGCACAC | ATGCTTCCAATTCTTTGTAGCA | B6 4bp > NOD |
| D3Mit41 | 3:97791322-97791530 | AATTTCTTCCTGTTACACTGAGCC | CATGAGAGAACTCCTTCCATCC | NOD 10bp > B6 |
| D3Mit213 | 3:98177336-98177481 | CCTTCTTTTTTTACTCTTCTACGTTG | GGTTCACAATTGCCAGTAACTG | B6 8bp > NOD |
| rs3158127 | 3:100649567-100649716 | TTAGTGGGACAGCGATCTGA | GCAGGCTGTTGCTACTTTCA | RFLP: SspI cuts B6 |
| rs3162098 | 3:100687102-100687285 | AACCTCTGAACGAGACAGCAA | GGCAAGGACAAGAAAGAGGA | RFLP: HindIII cuts NOD |
| rs221320455 | 3:101105076-101105273 | CACGCATTTCCATTGTCTTG | GAGTTGGCAGAGTTGGTGGT | B6 2bp > NOD |
| rs262915861 | 3:101145810-101145995 | GGAAGGATCCAGTGCCCTAT | GAACCTTGCATTCCTCAAGC | B6 12bp > NOD |
| rs243032810 | 3:101150943-101151103 | ACCTGGCTGCGTGTAAGTCT | AGTCAGAGGCAGGCTACCAA | NOD 2bp > B6 |
| rs38124092 | 3:122345852-122345966 | GCCCATATGATCCAATCACC | ATGGGTGGCATTATGGCTTA | RFLP: RsaI cuts NOD |
| D3Mit370 | 3:125522356-125522588 | CCTTTCTGATTATGTGGGCT | CCACTGAAGGATAACCACAG | NOD 18bp > B6 |
|  |  |  |  |  |
